# Molecular and phylogenetic characterization of the homoeologous *EPSP Synthase* genes of allohexaploid wheat, *Triticum aestivum* (L.)

**DOI:** 10.1186/s12864-015-2084-1

**Published:** 2015-10-23

**Authors:** Attawan Aramrak, Kimberlee K. Kidwell, Camille M. Steber, Ian C. Burke

**Affiliations:** Department of Crop and Soil Science, Washington State University, Pullman, WA USA; Wheat Genetics, Quality, Physiology and Disease Research, USDA-ARS, Pullman, WA USA

**Keywords:** Cloning, EPSP synthase, Glyphosate, Polyploid, *TaEPSPS-4A1*, *TaEPSPS-7A1*, *TaEPSPS-7D1*, *Triticeae* evolution, *Triticum aestivum*, Wheat

## Abstract

**Background:**

5-Enolpyruvylshikimate-3-phosphate synthase (EPSPS) is the sixth and penultimate enzyme in the shikimate biosynthesis pathway, and is the target of the herbicide glyphosate.  The *EPSPS* genes of allohexaploid wheat (*Triticum aestivum*, AABBDD) have not been well characterized. Herein, the three homoeologous copies of the allohexaploid wheat *EPSPS* gene were cloned and characterized.

**Methods:**

Genomic and coding DNA sequences of *EPSPS* from the three related genomes of allohexaploid wheat were isolated using PCR and inverse PCR approaches from soft white spring “Louise’.  Development of genome-specific primers allowed the mapping and expression analysis of *TaEPSPS-7A1*, *TaEPSPS-7D1*, and *TaEPSPS-4A1* on chromosomes 7A, 7D, and 4A, respectively. Sequence alignments of cDNA sequences from wheat and wheat relatives served as a basis for phylogenetic analysis.

**Results:**

The three genomic copies of wheat *EPSPS* differed by insertion/deletion and single nucleotide polymorphisms (SNPs), largely in intron sequences. RT-PCR analysis and cDNA cloning revealed that EPSPS is expressed from all three genomic copies. However, *TaEPSPS-4A1* is expressed at much lower levels than *TaEPSPS-7A1* and *TaEPSPS-7D1* in wheat seedlings. Phylogenetic analysis of 1190-bp cDNA clones from wheat and wheat relatives revealed that: 1) *TaEPSPS-7A1* is most similar to *EPSPS* from the tetraploid AB genome donor, *T. turgidum* (99.7 % identity); 2) *TaEPSPS-7D1* most resembles *EPSPS* from the diploid D genome donor, *Aegilops tauschii* (100 % identity); and 3) *TaEPSPS-4A1* resembles *EPSPS* from the diploid B genome relative, *Ae. speltoides* (97.7 % identity). Thus, *EPSPS* sequences in allohexaploid wheat are preserved from the most two recent ancestors. The wheat *EPSPS* genes are more closely related to *Lolium multiflorum* and *Brachypodium distachyon* than to *Oryza sativa* (rice).

**Conclusions:**

The three related *EPSPS* homoeologues of wheat exhibited conservation of the exon/intron structure and of coding region sequence, but contained significant sequence variation within intron regions. The genome-specific primers developed will enable future characterization of natural and induced variation in *EPSPS* sequence and expression.  This can be useful in investigating new causes of glyphosate herbicide resistance.

**Electronic supplementary material:**

The online version of this article (doi:10.1186/s12864-015-2084-1) contains supplementary material, which is available to authorized users.

## Background

5-Enolpyruvylshikimate-3-phosphate synthase (EC 2.5.1.19) or EPSP synthase (EPSPS) is the sixth and penultimate enzyme in the shikimate pathway [1]. EPSPS activity and the shikimate pathway are essential for the biosynthesis of the aromatic amino acids phenylalanine, tryptophan, and tyrosine, and for various secondary metabolites derived from the precursor chorismate, such as lignin, auxin, alkaloids, carotenoids, vitamins, and phenolic compounds [1, 2]. The synthesis of these compounds is essential to plant health and growth. EPSPS enzyme is potently inhibited by glyphosate, a non-selective herbicide widely used in weed management. A mutation in EPSPS is one mechanism conferring glyphosate resistance to weeds and crops. The amino acid substitution from proline at amino acid position 106 in EPSPS to serine, threonine, leucine, or alanine results in glyphosate resistance in weed species [3–5]. Thus, knowledge about the wheat *EPSPS* gene sequence, expression, exon-intron structure, and the development of genome-specific primers will be useful for examining natural and induced mutations in wheat *EPSPS*, as well as for detecting transgene contamination. For example, genome-specific primers and quantitative reverse transcription polymerase chain reaction (RT-qPCR) methods for measuring *EPSPS* mRNA levels are useful for determining if glyphosate resistance results from increased *EPSPS* expression levels, as previously described in *Dicliptera chinensis* (Chinese foldwing) [6]. The development of genome-specific primers may be useful to researchers investigating whether there are naturally occurring mutations in the wheat *EPSPS* genes if glyphosate resistant wheat is found in farmers’ fields. It is also interesting from the perspective of investigating wheat genome evolution based on the *EPSPS* sequence.

Currently, there is not a transgenic glyphosate-resistant wheat cultivar available for use by growers due in part to lack of acceptance by export markets. Common wheat (*T. aestivum*) or bread wheat is one of the major food grain crops belonging to the grass Poaceae family. The hybridization between the diploid AA genome-donor and the BB genome-donor generated the allotetraploid *T. turgidum* (AABB) or durum wheat that is currently grown for use in pasta [7–9]. The diploid A-genome donor is believed to have been *T. urartu* (A^u^A^u^), or a relative of the ancient crop plant einkorn *T. monococcum* (A^m^A^m^). The diploid B-genome donor is an unknown species (BB), but related to *Ae. speltoides* (SS). Subsequently, diploid *Ae. tauschii* (DD genome-donor) hybridized to tetraploid wheat, giving rise to the allohexaploid bread wheat (AABBDD). The large 17-gigabase allohexaploid wheat genome is comprised of 21 chromosomes (1*n*) with three sets of 7 chromosomes derived from each related (homoeologous) subgenome (A, B, and D) of the three diploid ancestors [7–10]. The 21 wheat chromosomes are designated based on the 7 homoeologous chromosome group and three genomes, such that we refer to chromosome 1A, 1B, 1D through chromosome 7A, 7B, and 7D. Allohexaploid wheat behaves like a diploid during meiosis such that chromosome 7A can only pair with 7A, not with 7B or 7D. The wheat genome has also evolved through translocation events [11]. For example, a segment of chromosome 7BS translocated to 4AL, and a segment of 4AL translocated to 5AL. Both gene cloning and gene expression analysis in wheat are complicated by the inherent genetic redundancy and by a large portion (more than 80 %) of non-coding DNA [8, 12]. Consequently, development of high quality genome-specific primers for wheat genes of interest is essential.

The EPSPS enzymes of different species are divided into two classes according to glyphosate sensitivity. The Class I EPSPS enzymes found in plants and bacteria (i.e. *Escherichia coli* and *Salmonella typhimurium*) are glyphosate sensitive and have been used to characterize the enzyme kinetics and active site [13]. The Class II EPSPS found in bacterial species including *Agrobacterium tumefaciens* sp. strain CP4, *Pseudomonas* sp. strain PG2982, and *Staphylococcus aureus* are glyphosate resistant and have been used to develop glyphosate-resistant crops [14]. The EPSPS of plants is nuclear-encoded but functions in the chloroplast. Thus, the premature EPSPS polypeptide contains an N-terminal transit peptide signal that is cleaved upon transport into the chloroplast [1].

Wheat EPSPS primers for PCR cloning based upon the *EPSPS* mRNA sequence of Chinese Spring wheat and upon wheat expressed sequence tag (EST) sequences were developed as part of this work. PCR is a simple and powerful technique used in molecular studies such as gene cloning, gene expression analysis, and disease diagnosis. PCR approaches have been successfully used in the isolation of homoeologous genes from hexaploid wheat and in quantification of gene expression from related genomes of hexaploid wheat [15–17]. The development of wheat genome-specific primers has been challenging due to: (i) the large portion of non-coding DNA in the wheat genome, (ii) the low level of polymorphism in the coding sequences of wheat homoeologues, and (iii) the lack of a reference genome sequence. The recent publication of the 0.6X draft genome sequence of Chinese Spring wheat and of the ‘Kukri’ wheat transcript assembly will help future wheat cloning efforts [18, 19].

Herein, we cloned the *TaEPSPS-7A1*, *TaEPSPS-7D1*, and *TaEPSPS-4A1* genes from the allohexaploid spring wheat cultivar ‘Louise’, as well as the homologous *EPSPS* cDNA sequences of four wheat progenitors. This allowed us to examine the evolution of the wheat *EPSPS* gene based on phylogenetic sequence analyses. The development of genome-specific *EPSPS* primers allowed us to examine the chromosome location of the wheat *EPSPS* genes and to compare the expression levels of the three homoeologous wheat *EPSPS* transcripts.

## Results and discussion

### Initial cDNA cloning revealed two major expressed homoeologous *EPSPS* genes

Preliminary experiments cloned wheat *EPSPS* cDNA sequences using primer pairs designed based on an alignment of two *T. monococcum* and thirteen *T. aestivum* (Chinese Spring) ESTs to amplify *EPSPS* sequence from all three wheat genomes (Table 1; Additional file 1). Cloning and sequencing of cDNA from allohexaploid wheat ‘Louise’ and from the wheat relatives *T. monococcum*, *Ae. speltoides*, *Ae. tauschii*, and *T. turgidum* was used to determine which primer pair could amplify the largest cDNA fragment from all wheat genomes and relatives. RT-PCR amplification with primer pairs F2-R2, F2-R3, and F2-R1 produced products from all lines but for *Ae. speltoides*, whereas F3-R2, F3-R3, and F3-R1 produced products from all genotypes including *Ae. speltoides*. The F3-R1 primer pair was selected for use in cDNA cloning because it produced the largest fragment, 1190-bp, containing the *EPSPS* coding region from exons 2 to 8 based on alignment with rice *EPSPS* [GenBank:AF413081].

**Table 1:** Primer sequences and primer positions based on *TaEPSPS-7A1*

^a^Primer No.	Primer name	^b^Sequence (5' to 3')	^c^Genome specificity	Primer position
*Forward*				
-	T7	TAATACGACTCACTATAGGG	-	-
1	F1.2	GCAATGGCGATGGCTG	**7A**	5'-UTR/exon 1
2	Int1_F2-D	AGCAGCATGTCCTGTTATCTTAT	**7D**	intron 1
3	Int1_F2-A/B	CAGCATGTCCTGTTCTCTTG	**4A**	intron 1
4	F2	AGGATGTCCACTACATGCTT	**7A**/7D	exon 2
5	F3	AGGATGTCCACTACATGCTC	**7D**/**4A**/7A	exon 2
6	F2-AB	GCAACTTATGTGCTTGATGGC	**7A**	exon 2/exon 3
7	InvF1-AB/D	GAGCGACCTATTGGTGACTTAG	**7D**/**7A**/4A	exon 3
8	InvF3-AB/D	CCACCTGTCCGTATCAACG	**7A**/7D/4A	exon 3
9	Int3_F1-AB	ATGTAATGCAACCTTAGACCGC	**7A**	intron 3
10	Int3_F1-A/B	CGATCAATGCGACCTTACACAAT	**4A**	intron 3
11	Int3_F1-D	GCAACGCGACCTTACACCGT	**7D**	intron 3
12	F19-AB	GGACAGATTCTACATTAAGGGAGGAC	**7D**/**7A**/4A	exon 4
13	Ex6_B-F	TACTTGAGATGATGGGAGCG	**4A**	exon 6
14	F13-AB	ATTTGGAAGGAAACACCTAAAA	**7A**/**4A**	exon 6
15	F14-D	GAAGGAAACACTTAAAGGCTGTC	**7D**	exon 6
16	F19-D	GTCCTGACACTTGTTCATTC	**7D/4A**	intron 6
17	F18-AB	GAACATCACGGCGATCGAC	**7A**/4A	exon 8
18	F18-D	GAACGTCACGGCGATCGAT	**7D**	exon 8
-	TaSEC_F	AGCAATTCGCACAATTATTACAAG	-	-
*Reverse*				
-	SP6	TATTTAGGTGACACTATAG	-	-
19	InvR2-AB-D	CAACAGGTTATCCACCACC	common	exon 2
20	InvR1-AB-D	GCATTGCAGTTCCAGCATTAC	common	exon 2
21	R1-AB	TTGTGCCAAGGAAACAATCGA	**7A**	exon 3
22	Ex4_R2	GCTCCGCAGTCACACCAAAAT	**7A**	exon 4
23	R2	TCAGAATGCTCCGCAGTCACA	**7A**/7D	exon 4
24	R3	TCAGAATGCGCCGCAGTCACG	**7D**/4A	exon 4
25	Ex4_R3	CTTGTATTTTTGTCCTCCCTTG	**4A**	exon 4
26	R19-AB	CCAGAATAATGGATGGCTCG	**7A**	intron 4
27	Ex6_B-R1	GCAACAACGGCAAGAGTCATT	**4A**	exon 6
28	R18-D	GACGCACGTGCTAGATTGCTTG	**7D**	intron 6
29	R12-AB	CGGATCGCGACCATTCTTTCA	**7A**	exon 7
30	R16-AB	TCTTTCGGGTGCATCCAGGG	**7A**	exon 8
31	R16-D	TCTTTCTGGTGCACCCCGGA	**7D**	exon 8
32	R1	CTAGTTCTTGACGAAGGTGCTTAG	common	exon 8
-	TaSEC_R	CTCACAGAAGACCTGGAAGC	-	-

^a^Primers are numbered in order based on location in *TaEPSPS-7A1* as shown in Additional file 4

^b^The start codon ATG and stop codon CTA are underlined

^c^Bold letters indicate genome-specific primers

**Fig. 1:**
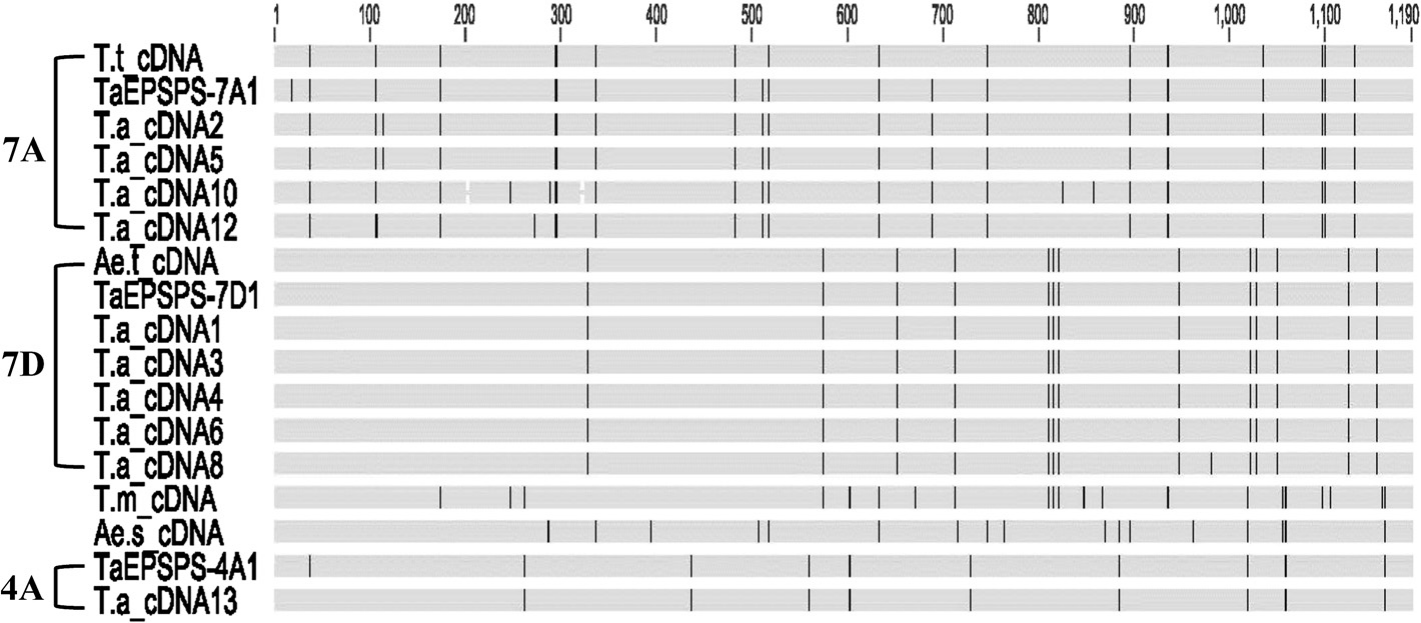
Nucleotide polymorphisms from an alignment of *EPSPS* cDNA sequences of allohexaploid wheat and wheat progenitors. Vertical lines in the diagram indicate the position of nucleotide polymorphisms identified in a ClustalW multiple sequence alignment of the 1190-bp cDNA clones from *T. aestivum* ‘Louise’ (T.a_cDNA#), with our 1190-bp cDNA consensus sequences for *T. turgidum* (AB progenitor) (T.t_cDNA), *Ae. tauschii* (D progenitor) (Ae.t_cDNA), *T. monococcum* (A-relative) (T.m_cDNA), and *Ae. speltoides* (B-relative) (Ae.s_cDNA). The equivalent 1190-bp sequence of the genomic DNA consensus sequences of “TaEPSPS-7A1, TaEPSPS-7D1, and TaEPSPS-4A1” are shown with intron sequences removed. Only the *TaEPSPS-7A1* and *TaEPSPS-7D1* cDNAs were recovered by F3-R1 PCR amplification. The corresponding 1190-bp *TaEPSPS-4A1* cDNA sequence (T.a_cDNA13) was derived from an independent experiment

The 1190-bp *EPSPS* cDNA clones from the wheat species above were sequenced and aligned. Consensus sequences were derived from eleven independent clones from allohexaploid wheat and from three independent clones from each wheat progenitor (Fig. 1; Additional file 2). The 1190-bp cDNA clones from the same species had 96−100 % nucleotide identity. Allohexaploid wheat was expected to have three functional copies of *EPSPS*, one each on the A, B, and D genomes [20]. The cDNA alignment revealed that there were two clusters of *EPSPS* cDNA sequences, one with high homology to the D genome donor *Ae. tauschii* (5 clones called “*EPSPS-D*”, 99.9−100 % sequence identity) and one with high homology to *T. turgidum* (4 clones called “*EPSPS-AB*”, 99.2−99.7 % sequence identity) (Additional file 2). The remaining two clones, T.a_cDNA9 and T.a_cDNA7 appeared to be chimeras, and may either be PCR artifacts or may represent *EPSPS*-related sequence from the wheat genome. The preliminary identification of the wheat *EPSPS-AB* and *EPSPS-D* cDNA sequences allowed the design of genome-specific primers using sequences containing multiple point mutation differences (Table 1; Additional files 1 and 3). Mapping later revealed that the *EPSPS-AB* primer pair F18-AB-R16-AB amplified *TaEPSPS-7A1* (*T. aestivum* 5-enolpyruvylshikimate-3-phosphate synthase) on chromosome 7A, and the *EPSPS-D* primer pair F18-D-R16-D amplified *TaEPSPS-7D1* on chromosome 7D (described below).

### Molecular cloning of a full-length genomic clone of *TaEPSPS-7A1*

**Fig. 2:**
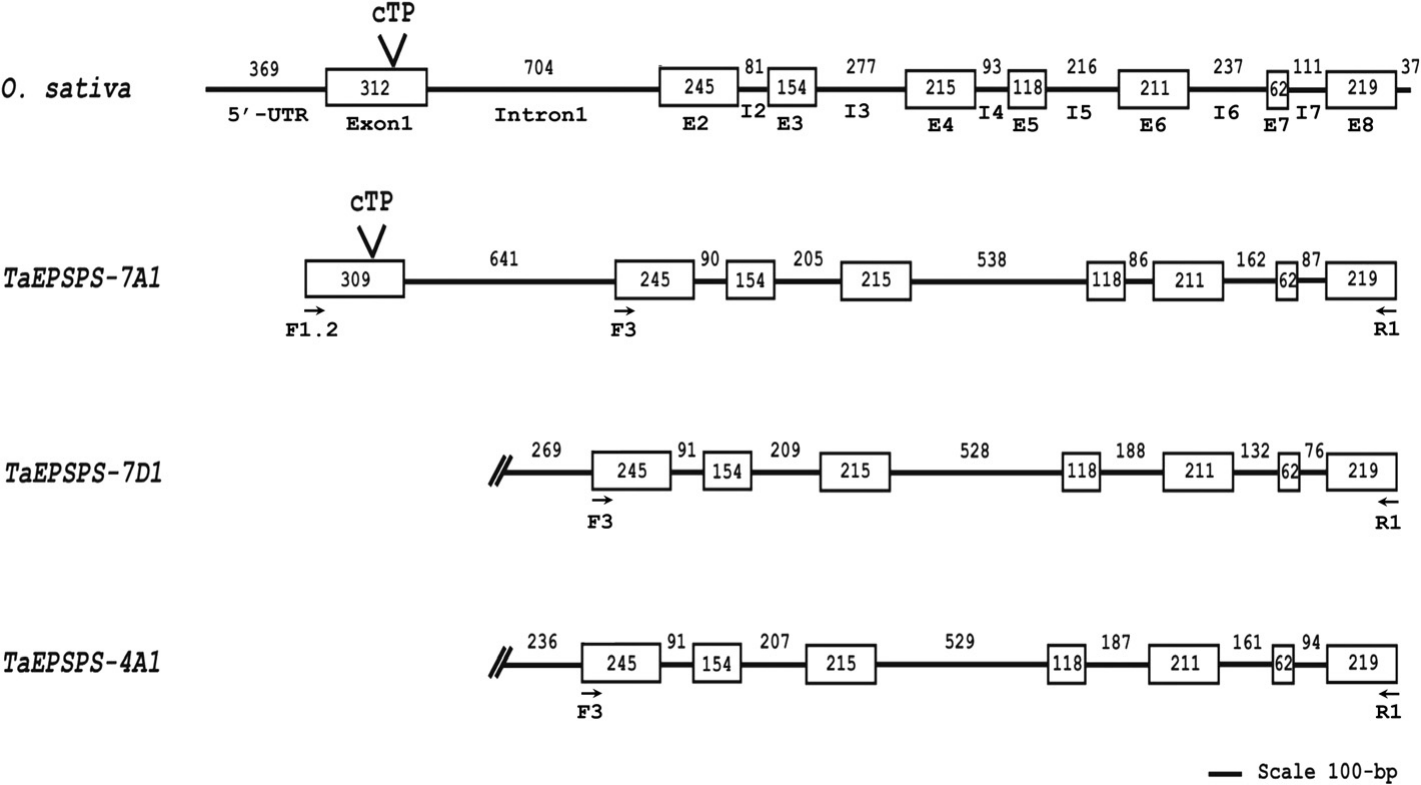
Exon-intron structure of the wheat *EPSPS* genes compared to *OsEPSPS*. The boxes and solid lines represent exons (E#) and introns (I#), respectively. Numbers indicate exon and intron size in bp. The cleavage site of the chloroplastic transit signal peptide (cTP) predicted by PredSL is shown. The positions of the primers F1.2, F3, and R1 (arrows) used for initial genomic and cDNA cloning are labeled

**Fig. 3:**
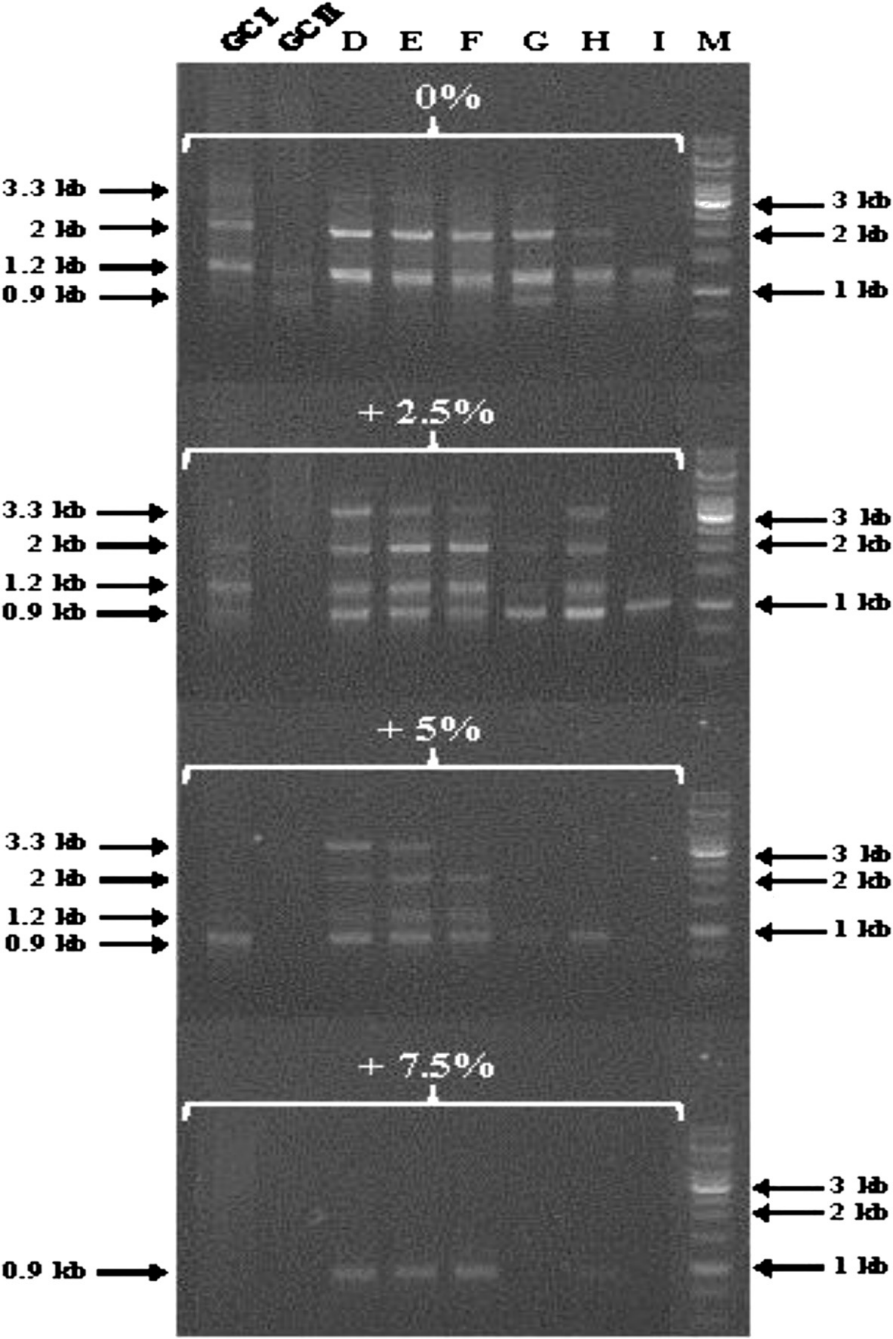
Optimization of the GC-rich *TaEPSPS-7A1* PCR amplification. Louise genomic DNA was amplified using the F1.2-R1 primer pair with the indicated buffers designed for amplification of difficult templates (GCI, GCII, D, E, F, G, H, and I), and the indicated concentrations of DMSO (0 %, 2.5 %, 5 %, and 7.5 %). ‘M’ stands for the 1 kb DNA ladder (Thermo Scientific)

In the cDNA cloning experiment, no primers upstream of F3 amplified the 5’ region of *EPSPS*, likely because it is highly GC-rich (~82 %). The primer F1.2 was designed to amplify this region upstream of F3 (Fig. 2). Eight different buffers designed to enhance the amplification efficiency of GC-rich targets were tested using the primer pair F1.2-R1 for PCR amplification of *EPSPS* from genomic DNA (see Methods; Fig. 3; Additional files 1, 3, and 4). Based on the rice *EPSPS* gene, the full length wheat *EPSPS* genomic clone was expected to be approximately 3.6-kb. PCR amplification with F1.2-R1 recovered 3.3-kb, 2-kb, 1.2 kb, and 0.9-kb products. Cloning and sequencing revealed that the 2-kb, 1.2-kb, and 0.9-kb products contained no homology to *EPSPS*. All of the four independent 3.3-kb clones contained a 3345-bp sequence with high identity (>99 %) to the *EPSPS-AB* cDNA clones. The 3345-bp consensus sequence, designated *TaEPSPS-7A1* [GenBank:KP411547], appeared to contain the full length wheat coding sequence based on alignment with rice *EPSPS* (*O. sativa EPSPS* or *OsEPSPS*) (Fig. 2). Based on alignment with the wheat *EPSPS-AB* cDNA and *OsEPSPS*, *TaEPSPS-7A1* is spliced into a 1533-bp cDNA encoding a predicted 510 amino acid protein with 87.3 % amino acid identity to OsEPSPS (Figs. 1 and 2; Table 2; Additional file 5).

**Table 2:** Percent homology of *EPSPS* sequences

Alignment levels	% Homology^a^				
	7A1/7D1^b^	7A1/4A1	7D1/4A1	7A1/Os	7A1/CP4
Genomic DNA sequence	84.7	85.7	93.8	62.3	39.3
cDNA sequence	96.3	96.6	97.4	83.5	41.4
Amino acid sequence	99.3	99.0	99.8	87.3	23.0

^a^Based on alignment of corresponding regions of exon 2 through exon 8

^b^Abbreviaitons: 7A = *TaEPSPS-7A1*; 7D = *TaEPSPS-7D1*; 4A = *TaEPSPS-4A1*; Os = *OsEPSPS* [GenBank:AF413081, AAL06593]; CP4 = *Agrobacterium* CP4 *EPSPS* [AB209952, BAD94823]

### Isolation of *TaEPSPS-7D1* and *TaEPSPS-4A1*

Polymorphic intron and exon sequences were used to develop genome-specific primers to clone and map the *TaEPSPS-7D1* and *TaEPSPS-4A1* genes (Table 1; Additional files 1 and 4) [21]. PCR amplification using Louise genomic DNA and the primer-pair combinations F13-AB-R1, F14-D-R1, and F3-R2 recovered *EPSPS* sequences containing polymorphisms compared to *TaEPSPS-7A1*. The F14-D-R1 primer pair amplified 600-bp and 838-bp, F13-AB-R1 amplified a 647-bp, and F3-R2 amplified a 838-bp product. The 600-bp (F14-D-R1) and 838-bp (F3-R2) products shared exon regions with 100 % identity to the 1190-bp *EPSPS-D* and of *Ae. tauschii* cDNA sequences (Table 1; Additional files 1, 2, and 4). Thus, two partial *TaEPSPS-7D1* genomic clones were identified. Cloning and sequencing revealed that the 647-bp (F13-AB-R1) product was a partial genomic clone of *TaEPSPS-4A1* based on lack of homology to *TaEPSPS-7A1* and the *TaEPSPS-7D1*. Attempts to amplify the 5’ genomic region of the *TaEPSPS-7D1* and *TaEPSPS-4A1* open reading frame (ORF) using combinations of F1.2 and several reverse primers designed to be 4A- and 7D-specific based on intronic sequences failed to recover a product, suggesting that the F1.2 primer is specific to *TaEPSPS-7A1* (data not shown).

Nested inverse-PCR (IPCR) strategies were devised to clone the remaining genomic sequences of *TaEPSPS-7D1* and *TaEPSPS-4A1* (Additional files 1 and 4) [22, 23]. Primers were developed for the D genome copy based on the 600-bp (F14-D-R1) *TaEPSPS-7D1* clone, and for the *TaEPSPS-4A1* based on the 647-bp product. *TaEPSPS-7D1* genomic sequence was recovered from F18-D-R18-D amplification of circularized *Acc*I-digested genomic DNA. The resulting 1261-bp clone included 1114-bp of *TaEPSPS-7D1* plus 147-bp of 7D chromosome sequence flanking the 3’end of the *TaEPSPS-7D1* gene. InvF3-AB-InvR2-AB amplification of the *Hind*III- and *Acc*I-digested DNA template recovered 702-bp and 682-bp products, respectively. The 702-bp product overlapped with the *TaEPSPS-7D1* genomic sequence, whereas the 682-bp (*Acc*I) fragment contained *TaEPSPS-4A1* sequences.

Genome-specific primers were designed for PCR amplification of longer genomic clones of the *TaEPSPS-4A1* (Int1_F2-A/B-R1 amplified the 2597-bp clone, Int3_F1-A/B-R1 the 1918-bp clone) and *TaEPSPS-7D1* (Int1_F2-D-R1 amplified the 2554-bp clone, Int3_F1-D-R1 the 1868-bp clone). Sequencing of these clones in both directions recovered the missing genomic sequences of both *TaEPSPS-7D1* and *TaEPSPS-4A1* (Additional files 1 and 4). Thus, we recovered the genomic sequences of *TaEPSPS-7D1* [GenBank:KP411548] and of *TaEPSPS-4A1* [KP411549] including a partial sequence for intron 1 and complete sequences for the remaining introns and for exons 2 through 8.

### Determining the chromosome location of the wheat *EPSPS* genes

**Fig. 4:**
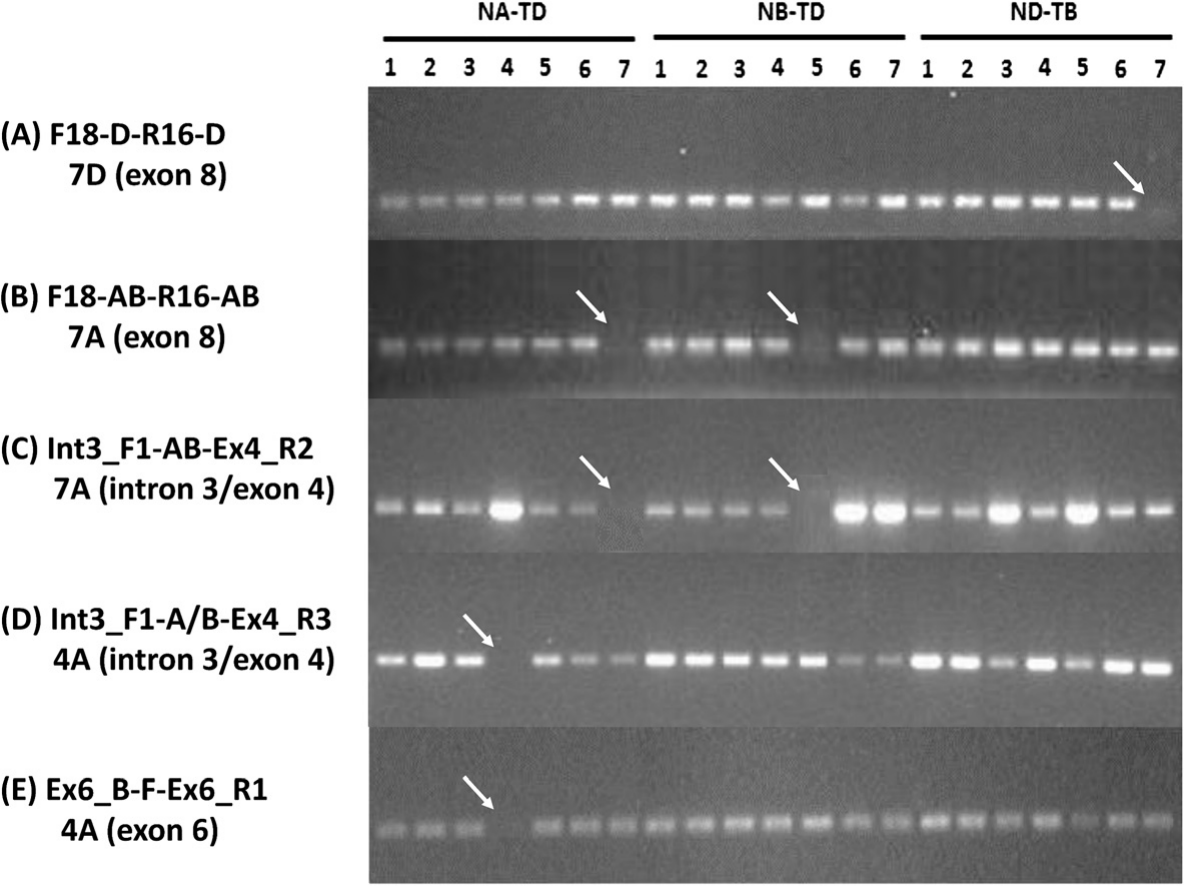
Mapping the chromosome locations of the wheat *EPSPS* genes using nulli-tetrasomic lines. PCR amplification was performed using indicated Chinese Spring nulli-tetrasomic genomic DNA. These lines contain no copy of one chromosome (nullisomic), and 4 doses (tetrasomic) of a homoeologous chromosome. The lines are referred to as nulli1A-tetra1D or N1A-T1D, N2A-T2D, etc. DNA was amplified with the indicated primer pairs for: **a** *TaEPSPS-7D1* failed to amplify in N7D-T7B line with primers F18-D-R16-D indicating localization to chromosome 7D; (**b** and **c**) *TaEPSPS-7A1* failed to amplify in N7A-T7D with indicated primers; (**d** and **e**) *TaEPSPS-4A1* failed to amplify in N4A-T4D

PCR amplification of genomic DNA from nulli-tetrasomic Chinese Spring wheat lines was used to determine the chromosome location and to confirm primer specificity during the cDNA and genomic cloning of *TaEPSPS-7A1*, *TaEPSPS-4A1*, and *TaEPSPS-7D1* (see Methods) [16]*.* For mapping, PCR amplification of nulli-tetrasomic genomic DNA was performed using gene-specific primers (Fig. 4; Additional file 1). The location of each *EPSPS* copy was defined based on failed PCR amplification in a specific nulli-tetrasomic line. Primers for the *TaEPSPS-7D1* failed to show amplification using the nulli7D-tetra7B (no copies 7D, 4 copies 7B) as the genomic DNA template. Similarly, the *TaEPSPS-4A1* and *TaEPSPS-7A1* primers failed to amplify using the nulli4A-tetra4D (no copies 4A, 4 copies 4D) and the nulli7A-tetra7D (no copies 7A, 4 copies 7D) as template, respectively. Thus, each wheat *EPSPS* clone was identified based on its chromosome location on 7A, 4A, and 7D, respectively.

Homoeologous genes of common wheat are usually located on the same group of chromosomes. For example, the wheat *PDI* (protein disulfide isomerase) homoeologues positioned on group 4 (4A, 4B, 4D), *DMC1* (RecA protein) on group 5, and *RAD51* (RecA protein) and *SSII* (starch synthase II) on group 7 [15, 17, 21]. The three wheat *EPSPS* genes belong to homoeologous group 7, since the allohexaploid wheat genome contains a chromosomal translocation from chromosome 7BS onto 4AL that occurred in the tetraploid ancestor [11]. We identified clones with high identity to *TaEPSPS-7A1*, *TaEPSPS-7D1*, and *TaEPSPS-4A1* on chromosomes 7AS, 7DS, and 4AL, respectively, in the 0.6X draft Chinese Spring wheat genome sequence [18]. This confirmed the chromosomal location of the wheat *EPSPS* genes.

We also observed that the *TaEPSPS-7A1* primers failed to amplify a PCR product from the nulli5B-tetra5D line (no copies 5B, 4 copies 5D) (Fig. 4b and c). No homologue of *TaEPSPS-7A1* was identified on chromosome 5B of the 0.6X Chinese Spring draft sequence. Moreover, there could not be a copy of *TaEPSPS-7A1* on chromosome 5B because such a duplication would resulted in presence of a *TaEPSPS-7A1* PCR product in both the nulli7A-tetra7D and nulli5B-tetra5D lines. Thus, it appears that the nulli5B-tetra5D line contained a deletion of the *TaEPSPS-7A1* gene. Consistent with this reasoning, a previous study of chromosome aberrations in nulli-tetrasomic lines showed that the nulli5B-tetra5D line contains a deletion in the chromosome 7AS region that likely contains *TaEPSPS-7A1* [24].

The cloning experiments described here were initiated before publication of the 0.6X Chinese Spring draft sequence, and provide new sequence and expression information [18]. The Chinese Spring *TaEPSPS-7A1*, *TaEPSPS-7D1*, and *TaEPSPS-4A1* genomic DNA contigs were incomplete (see Contigs 7AS_59852, 7AS_ 4136845, 7DS_3942167 and 7DS_3891840, and 4AL_7136532 and 4AL_7168802). They included a partial sequence of intron 1 and genomic sequence downstream of intron 1, but lacked the GC-rich exon 1 sequence recovered here for *TaEPSPS-7A1* from Louise spring wheat. IWGSC recovered two overlapping cDNA clones (1158-bp) with 95.4−99.8 % similarity to *TaEPSPS-7A1*, but did not recover cDNA clones for *TaEPSPS-7D1* and *TaEPSPS-4A1*. The *TaEPSPS-7D1* and *TaEPSPS-4A1* cDNA clones recovered in the current study proved that these genes are expressed.

### Recovery of cDNA sequence of *TaEPSPS-4A1*

The cDNA clones of *TaEPSPS-7A1* and *TaEPSPS-7D1*, but not of *TaEPSPS-4A1*, were recovered from total seedling RNA by RT-PCR amplification with the F3-R1 primer pair. Based on the genomic sequence, the F3-R1 primers should also amplify the *TaEPSPS-4A1* cDNA. To determine whether *TaEPSPS-4A1* is a pseudogene or a low-abundance transcript, we attempted to clone the *TaEPSPS-4A1* cDNA using gene-specific primer pairs. F3-Ex6_B-R1 and Ex6_B-F-R1 amplified 874-bp and 470-bp products respectively, using ‘Louise’ cDNA as a template (Table 1; Additional file 1). Five independent clones from each product were sequenced. All 470-bp clones had 100 % identity to the *TaEPSPS-4A1* exon sequences*.* The F3-Ex6_B-R1 primers were less *TaEPSPS-4A1*-specific, since one 874-bp clone corresponded to *TaEPSPS-4A1*, three to *TaEPSPS-7D1*, and one to *TaEPSPS-7A1*. The complete 1190-bp *TaEPSPS-4A1* cDNA sequence was derived from the two overlapping 874-bp and 470-bp clones (T.a_cDNA13 in Fig. 1; Additional file 1). Thus, *TaEPSPS-4A1* may be expressed at lower levels than *TaEPSPS-7A1* and *TaEPSPS-7D1* in seedlings.

### Expression levels of the three wheat *EPSPS* homoeologues

**Fig. 5:**
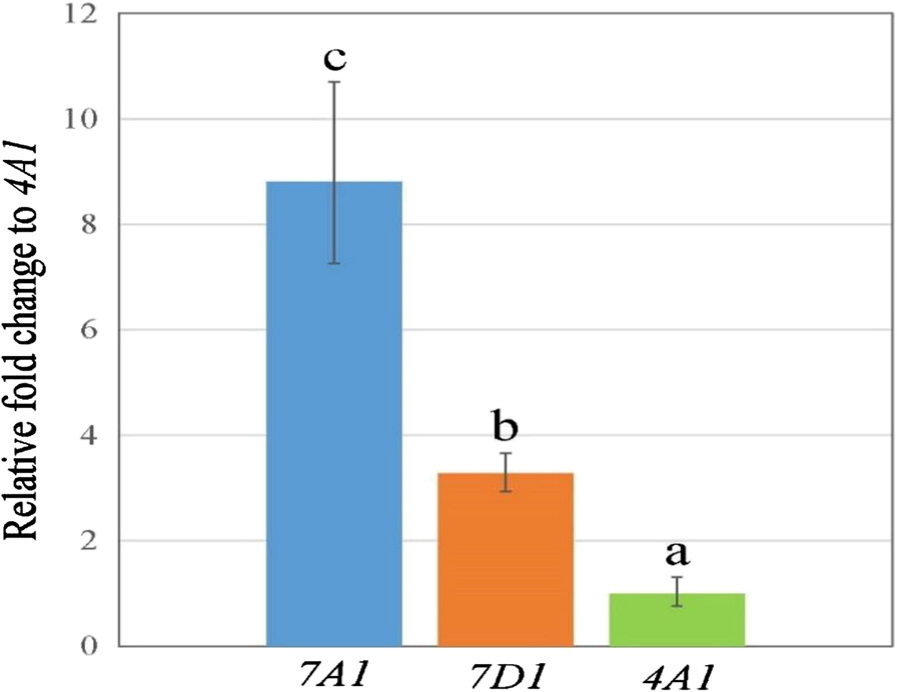
RT-qPCR analysis of wheat *EPSPS* gene expression in seedlings. The relative expression levels of *TaEPSPS-7A1* (*7A1*), *TaEPSPS-7D1* (*7D1*), and *TaEPSPS-4A1* (*4A1*) were determined by RT-qPCR using genome-specific primers (Additional file 1). The fold change was calculated relative to the *TaEPSPS-4A1* mRNA level (set to 1) using the comparative C_q_ method. Letters (a, b, and c) represent significant differences (*P* < 0.005, based on a one-way ANOVA with Tukey’s adjustment at the 99 % significance level). Bars indicate the standard error

We examined the relative expression levels of *TaEPSPS-7A1*, *TaEPSPS-7D1*, and *TaEPSPS-4A1* in seedlings using RT-qPCR analysis*.* Expression was examined at the 3 to 5-leaf stage because glyphosate is typically foliar-applied to young seedlings, and because *EPSPS* expression can be higher in leaves than in roots [25]. RT-qPCR analysis revealed that all three wheat *EPSPS* transcripts were actively expressed, but accumulated at different levels (Fig. 5; Additional file 1). The *TaEPSPS-7A1* transcript was most highly expressed, showing 3-fold higher expression than *TaEPSPS-7D1* and almost 9-fold higher expression than *TaEPSPS-4A1* (P-value < 0.001). The *TaEPSPS-4A1* mRNA levels were significantly lower than *TaEPSPS-7D1* (*P*-value = 0.003). The relatively low *TaEPSPS-4A1* mRNA levels were consistent with the difficulty recovering the *TaEPSPS-4A1* cDNA using the F3-R1 primer pair that was able to bind all three transcripts.

The differential expression of *TaEPSPS-7A1*, *TaEPSPS-7D1*, and *TaEPSPS-4A1* may have evolved after the polyploidization event. Previous studies have examined wheat gene expression in synthetic allohexaploid wheat generated through man-made polyploidization events. In such synthetic allohexaploids, as many as 87 % of wheat genes were expressed at similar levels from the A, B, and D genomes, although sometimes there was expression predominantly from one or two homoeologues (2.9 %) [26, 27]. A homoeologue may be lost through deletion or may become silenced during wheat genome evolution [20, 27]. Moreover, differential regulation may result from evolution of regulatory mechanisms through mutations in promoters or enhancer regions [26, 28, 29].

### Sequence comparison and gene structure

Cloning of the cDNA and genomic clones for *TaEPSPS-7A1*, *TaEPSPS-7D1*, and *TaEPSPS-4A1* allowed us to compare the sequence and intron/exon structures of the three wheat *EPSPS* homoeologues (Fig. 2; Additional file 3). The complete *TaEPSPS-7A1* coding sequence (7 introns/8 exons) was cloned, including the 3342-bp ORF, and the 1533-bp cDNA encoding a predicted 510 amino acid protein (53.8 kDa). The *TaEPSPS-4A1* (2729-bp) and *TaEPSPS-7D1* (2717-bp) genomic clones included all intron and exon sequences except exon 1 and a portion of intron 1. Based on the 1190-bp cDNA and incomplete genomic sequences of *TaEPSPS-7D1* and *TaEPSPS-4A1*, we can derive partial 1224-bp cDNA sequences containing exons 2 through 8, but not exon 1. The 1224-bp cDNA partial sequence encodes a predicted 407 amino acid EPSPS enzyme (43.4 kDa TaEPSPS-4A1 and 43.5 kDa TaEPSPS-7D1) (Additional file 5). The wheat EPSPS proteins show higher homology to the glyphosate-sensitive Class I EPSPS of rice (87.3 % identity) than to the glyphosate-resistant Class II EPSPS of CP4 (23 % identity) (Table 2) [25].

Based on previous characterization of *EPSPS* in *Amaranthus palmeri*, *Petunia hybrida*, and *Arabidopsis thaliana*, we expected exon 1 to contain the chloroplastic signal peptide [30, 31]. EPSPS is a nuclear-encoded protein that must be transported to the chloroplast. The chloroplastic transit signal peptide of the EPSPS protein sequence is cleaved once the protein is located in the chloroplast [1]. PredSL was used to search for the expected chloroplastic signal peptide [32]. A chloroplastic signal peptide was detected in exon 1 of TaEPSPS-7A1 (Additional file 6). These signal peptide amino acid sequences were highly conserved in other grasses including *B. distachyon*, *O. sativa*, *Sorghum halepense*, and *Zea mays*. Based on the cleavage point for the chloroplastic signal peptide, the cloned regions of the TaEPSPS-4A1 and TaEPSPS-7D1 include all but the first 32 amino acids of the enzyme.

The gene structure of each *EPSPS* copy was originally predicted using NetGene2 and Neutral Network splice-junction analysis programs [33, 34], and then confirmed by comparing genomic and cDNA sequences (Fig. 2). When the genomic and cDNA sequences were aligned using the Geneious™ Global Alignment tool, the actual splice junctions were identical to those predicted and to those of rice and *A. palmeri* [GenBank:AF413081, FJ861242.1] [30, 35]. There was a higher frequency of SNP and In/Del variation within intron than exon sequences among the three wheat *EPSPS* sequences (Fig. 1; Additional file 3). At the genomic DNA level, *TaEPSPS-7D1* had higher homology to *TaEPSPS-4A1* (93.8 % identity) than to *TaEPSPS-7A1* (84.7 % identity) (Table 2). The three wheat *EPSPS* homoeologues were very similar, with more than 96 % and 99 % identity at the cDNA and amino acid levels, respectively.

### Phylogenetic analysis of the *EPSPS* genes of allohexaploid wheat

**Fig. 6:**
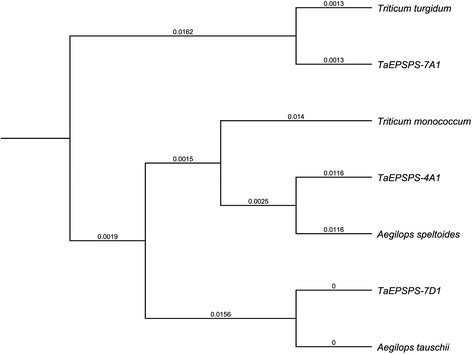
Phylogenetic tree of *EPSPS* genes of wheat and wheat relatives. The UPGMA method was used to analyze nucleotide sequence similarity based on the 1190-bp cDNA sequences. Numbers at each branch indicate the genetic distance

Phylogenetic analyses of wheat *EPSPS* were conducted using the 1190-bp cDNA sequences of *TaEPSPS-7A1*, *TaEPSPS-7D1*, and *TaEPSPS-4A1*, and of the wheat relatives *T. turgidum* [GenBank:KR559878] (AABB), *T. monococcum* [KR559879] (A^m^A^m^, A-relative), *Ae. speltoides* [KR559880] (SS, B-relative), and *Ae. tauschii* [KR559881] (DD) (Figs. 1 and 6; Additional File 2). Note that only a single consensus sequence was derived from the tetraploid *T. turgidum.* The *TaEPSPS-7D1* cDNA clone was in the same clade as *Ae. tauschii*, consistent with evidence that the D-genome was incorporated into allohexaploid wheat fairly recently [9]. *TaEPSPS-4A1* was most closely related to the B-genome relative *Ae. speltoides*, consistent with our interpretation that *TaEPSPS-4A1* is the B-genome copy of *EPSPS*, likely located within the 7BS translocation to chromosome 4AL. Interestingly, *EPSPS* from the A-genome relative *T. monococcum* is more closely related to *TaEPSPS-4A1* than to *TaEPSPS-7A1. TaEPSPS-7A1* was very closely related to the cDNA cloned from tetraploid *T. turgidum* (0.0026 genetic distance).

Phylogenetic analysis indicated that the *TaEPSPS-7A1* and *TaEPSPS-7D1* genes are most closely related to those of the most recent progenitors, tetraploid *T. turgidum* (99.7 % identity) and diploid *Ae. tauschii* (100 % identity), respectively. *TaEPSPS-7A1* is more closely related to the *EPSPS* cloned from *T. turgidum* (AABB) than from the A-genome donor relative *T. monococcum* (96.1 % identity) or the B-genome relative *Ae. speltoides* (97.1 % identity). *TaEPSPS-4A1*, however, is more closely related to the B-genome relative *Ae. speltoides* (97.7 % identity) than to *T. turgidum* (96.8 % identity), and is consistent with previous analyses concluding that homoeologues of *T. aestivum* (AABBDD) are more closely related to homologues in *Ae. tauschii* (DD) and *T. turgidum* (AABB) than to the primitive A- and B-genome ancestors [7, 21]. It appears that the diploid A- and B-genome progenitors of allohexaploid wheat have evolved independently from *T. turgidum* following the tetraploidization event, whereas the D-genome donor *Ae.* t*auschii* is still very closely related to the D-genome of *T. aestivum* [reviewed by 27]. Moreover, *T. monococcum* has evolved independently from *T. turgidum* since its last shared ancestor with the wild einkorn A-genome progenitor *T. urartu*. The genetic variation in the wheat A- and B-genome *EPSPS* homoeologues appears to have resulted from the accumulation of point mutations, small DNA insertions/deletions, and genetic recombination, likely following the polyploidization event (Fig. 1; Additional files 2 and 3) [reviewed in 9, 27].

**Fig. 7:**
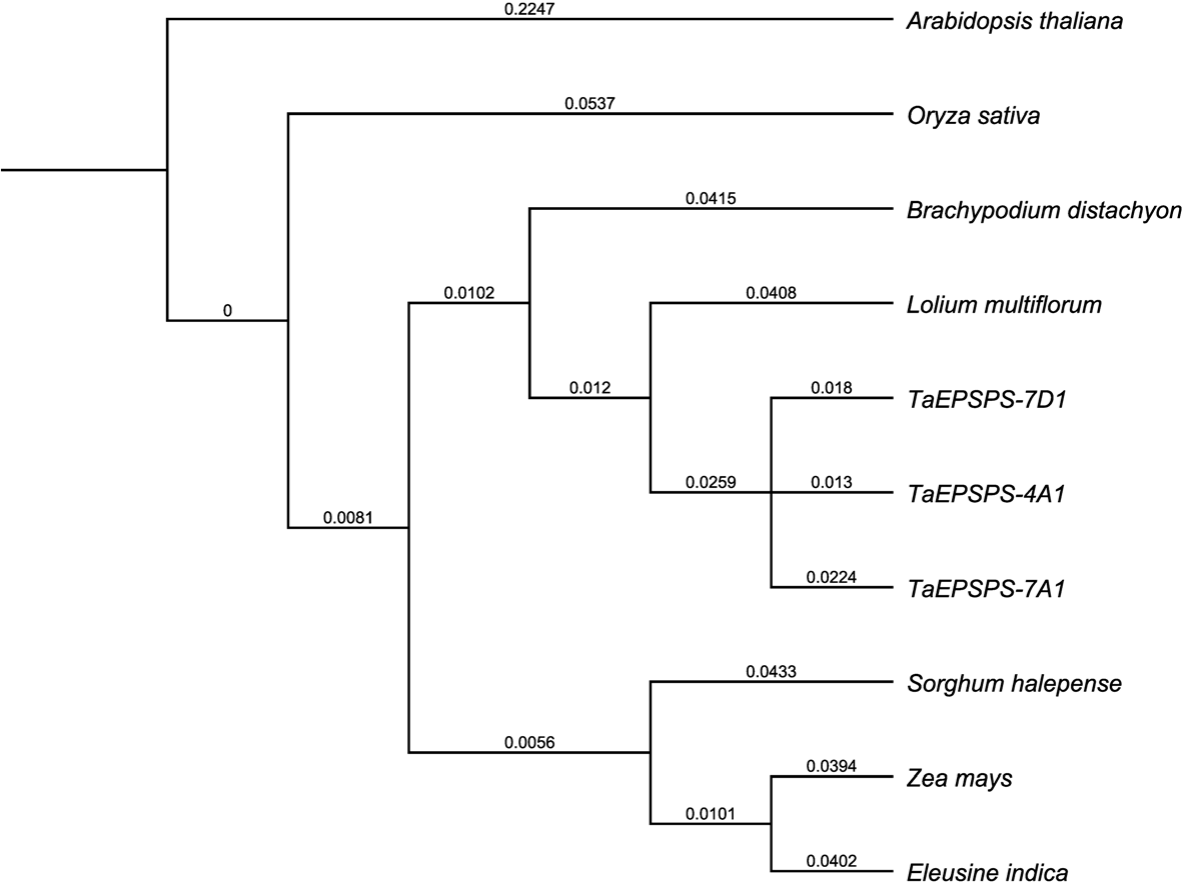
Phylogenetic tree of *EPSPS* genes of grass species. The 1190-bp cDNA sequences of *TaEPSPS-7A1*, *TaEPSPS-4A1*, and *TaEPSPS-7D1* from allohexaploid wheat ‘Louise’ were compared to the corresponding *EPSPS* sequences from other species using the Neighbor-Joining method with the dicot *A. thaliana* as the outgroup. Numbers at each branch represent the genetic distance. The *EPSPS* homologues include *A. thaliana* [GenBank:NM_130093], *B. distachyon* [XM_003557194], *E. indica* [HQ403647], *S. halepense* [HQ436353], *Z. mays* [X63374], *O. sativa* [AF413081], and *L. multiflorum* [DQ153168]

The phylogenetic relationship of the three wheat *EPSPS* 1190-bp cDNA sequences was also examined compared to the corresponding sequence in other grasses using the dicot *A. thaliana* as the outgroup (Fig. 7). The three *TaEPSPS* genes were most closely related to *EPSPS* of *L. multiflorum* and *B. distachyon* and more distantly related to *EPSPS* of rice. The remaining clade includes monocots *Z. mays*, *S. halepense*, and *Eleusine indica*.

Allohexaploid wheat, *L. multiflorum*, and model plant *B. distachyon* are believed to have originated from a common ancestor that diverged from rice about 46 million years ago (MYA), whereas the grasses in general are believed to have shared a common ancestor about 90 MYA [36, 37]. A comparative genome analysis between wheat and *B. distachyon* suggested that these two species diverged at 32–39 MYA [38]. Moreover, analysis of acetyl-CoA carboxylase (*ACCase*) and 3-phosphoglycerate kinase (*PGK*) genes between wheat and ryegrass (*L. rigidum*) revealed that they diverged about 35 MYA [7].

## Conclusions

The study reports the cloning, sequencing, and mapping of three homoeologous *EPSPS* genes named *TaEPSPS-7A1* [GenBank:KP411547], *TaEPSPS-7D1* [KP411548], and *TaEPSPS-4A1* [KP411549] from allohexaploid wheat cultivar ‘Louise’. The genes were located on chromosomes 7AS, 4AL (translocated from 7BS), and 7DS, respectively. The comparison of cDNA and genomic DNA sequences allowed confirmation of the intron/exon structure, as well as the development of genome-specific primers. RT-qPCR analysis revealed that all three *EPSPS* homoeologues are actively transcribed. However, *TaEPSPS-7A1* had the highest level of expression in young leaves. The three *EPSPS* genes had a high degree of sequence conservation at both the cDNA and protein levels. Phylogenetic analysis suggests that the *EPSPS* of wheat is closely related to *EPSPS* of *L. multiflorum* and *B. distachyon*.

## Methods

### Plant material and growth conditions

Grains of *T. aestivum* soft white spring ‘Louise’ (AABBDD, Reg. No. CV-987, PI 634865) [39] and of wheat relatives [*T. turgidum* ssp. dicoccoides (AABB), *T. monococcum* DV92 (A^m^A^m^, A-relative) [40], *Ae. speltoides* (SS, B-relative), and *Ae. tauschii* (DD)] were grown in a greenhouse at 21−24 °C daytime and 15−17 °C nighttime with a 16-h day/8-h night photoperiod (Wheat Growth Facility, Washington State University, Pullman WA). At the 3 to 5-leaf stage, young leaf tissue was harvested, submerged in liquid nitrogen, and stored at -80 °C. Frozen leaf tissue was ground to a fine powder with a cold mortar and pestle under liquid nitrogen and stored at -80 °C until use for nucleic acid extraction.

### Nucleic acid isolation and cDNA synthesis

Louise genomic DNA was isolated from 100 mg of ground leaf tissue using the sodium bisulfite method [41, 42]. Total RNA was extracted using the Trizol reagent (Invitrogen) method according to the manufacturer’s protocol. Before performing RT-qPCR, RNA samples were treated with 2 U of DNase I to prevent DNA contamination using the DNA-free RNA kit™ (Zymo Research). Nucleic acid concentration and quality were assessed by NanoDrop™ UV spectrophotometry and by agarose gel electrophoresis. For the cDNA cloning experiment, first-strand cDNA was synthesized from 5 μg of total RNA using the ImProm-II™ reverse transcription system kit (Promega) according to the manufacturer’s instructions. For the RT-qPCR experiment, first-strand cDNA was synthesized from 1 μg of total RNA using the ProtoScript® first strand cDNA synthesis kit (NEB) following the manufacturer’s instructions. Synthesized cDNA was adjusted to a final concentration of 100 ng/μl for cDNA cloning and to 2 ng/μl with RNase-free water for RT-qPCR experiments.

### Primer design

Primers designed to either amplify all copies or a specific copy of the *TaEPSPS* cDNA were based on a sequence alignment of a *T. aestivum* Chinese Spring cDNA consensus sequence [GenBank: EU977181], with *T. aestivum* ESTs [DR740760, CJ674666, CJ962197, CJ567699, CJ565347, CJ9534710, CJ653434, BQ743545, BG605080, BF483288, CD889643, and EB513993], and *T. monococcum* ESTs [BF200451 and BF199999] (Table 1). Genome-specific primers used to amplify both cDNA and genomic DNA were based on cDNA alignments of the 1190-bp cDNA clones from Louise wheat, and from the wheat relatives *T. monococcum*, *T. turgidum*, *Ae. speltoides*, and *Ae. tauschii* (Table 1; Additional file 2). Both primers used for gene cloning and genome-specific primers used for mapping and RT-qPCR were derived from alignments of cDNA and genomic DNA sequences of *TaEPSPS-7A1*, *TaEPSPS-7D1*, and *TaEPSPS-4A1* (Table 1; Additional files 1, 3 and 4). The genome-specificity of primers was checked using amplification of cDNA from wheat progenitors and genomic DNA from nulli-tetrasomic lines.

### PCR and IPCR amplification

PCR amplification was performed as follows: 1) 1 cycle of 94 °C for 5 min; 2) 35 cycles of 94 °C for 1 min, annealing for 20–40 s, and extension at 72 °C for 1 min/kb, and 3) 1 cycle of 10 min at 72 °C for the final extension. The optimized annealing temperatures and the extension times are provided in Additional file 1. Unless otherwise indicated, PCR reactions generally contained: 1) 50–60 ng gDNA or 200 ng cDNA template, 2) 0.2 mM dNTPs, 2 mM MgCl_2_, 3) 0.4 μM of each primer, 4) 1X Mango buffer (Bioline), and 5) 0.5 U of either Mango *Taq* enzyme (Bioline) or of the proofreading enzymes *LA Taq* or *Ex Taq* (TaKaRa) depending on the application.

PCR amplification of the GC-rich 5’-region (exon 1) of the *TaEPSPS-7A1* gene were optimized using a panel of buffers designed for amplification of difficult templates (Fig. 2; Additional file 3). The PCR reactions contained 0.2 μM of F1.2 and R1 primers, 1 U proofreading *Ex Taq* DNA polymerase (TaKaRa), and 1X each of the following premixed commercial buffers: GCI and GCII with 2 mM MgCl_2_ (TaKaRa), and Buffers D, E, F, G, H, and I (Epicentre™). The reactions were also supplemented with DMSO at 0 %, 2.5 %, 5 %, and 7.5 % in each buffer [43, 44]. The 3.3-kb *TaEPSPS-7A1* clone was successfully recovered using buffer D and 5 % DMSO, and sequenced in both the forward and reverse directions (Table 1).

Inverse PCR (IPCR) was used to clone unknown sequences of *TaEPSPS-7D1* and *TaEPSPS-4A1* genes (Table 1; Additional files 1 and 4). IPCR was performed using, 1.5 μg of Louise gDNA digested with restriction enzymes *Hind*III and *Acc*I (10 U, Thermo Scientific) at 37 °C for 2 h. After heat inactivation at 80 °C for 20 min, DNA fragments were gel-purified using the QIAEX II gel extraction kit. The DNA fragments were diluted to 4 ng/μl then self-ligated into circular DNA by incubating with T4 DNA ligase (NEB Biolabs) at 16 °C for 16 h. The circularized DNA was again gel purified and diluted to 10 ng/μl. Genomic DNA without enzyme digestion served as a control for detecting artifactual fragments. The first round of IPCR was carried out in 20 μl of reaction mixture containing 40 ng of circularized gDNA, 0.4 μM of primers InvF1-AB/D-InvR1-AB/D for *TaEPSPS-4A1* or F19-D-R18-D for *TaEPSPS-7D1*, 0.2 mM dNTPs, 2 mM MgCl_2_, 1X GCI buffer (TaKaRa), and 1 U *LA Taq* polymerase (TaKaRa). One microliter of the first round PCR reaction was used as a template in the second round of IPCR using the same conditions but for substitution of 1 U Mango *Taq* DNA polymerase and buffer (Bioline) for the enzyme and use of the primers InvF3-AB/D-InvR2-AB/D for *TaEPSPS-4A1* and F18-D-R18-D for *TaEPSPS-7D1*.

### DNA cloning and sequencing

In order to obtain multiple independent clones, the PCR and IPCR reactions were aliquoted into at least 3 separate tubes before performing PCR. PCR products were separated and visualized on a SYBR® Safe (Invitrogen)-stained 0.8 to 2 % agarose gel depending on the expected amplicon size. For cloning, gel-excised DNA fragments were purified using the QIAEX II gel extraction kit (Qiagen), and ligated into the pGEM-T easy vector (Promega). Ligation reactions were transformed into *E. coli*, and plasmids with insertion were selected by blue-white screening and confirmed by PCR. Clones were then isolated for the plasmids. Plasmid DNA clones prepared using the QIAprep miniprep kit (Qiagen) were sequenced in both the forward and reverse directions using either Elim Biopharmaceuticals, Inc. (Hayward, CA) or the Molecular Biology and Genomics Core (WSU, Pullman) sequencing services.

### Mapping the location of *EPSPS* genes in hexaploid wheat

PCR amplification using genomic DNA of nulli-tetrasomic lines (NA-TD, NB-TD, and ND-TB) in Chinese Spring wheat was used to check primer specificity and to determine which *EPSPS* gene was located on which wheat chromosome (Table 1; Additional file 1). Nulli-tetrasomic Chinese Spring genotypes contain four copies of one chromosome pair (tetrasomic) to compensate for lack of a homoeologous chromosome pair (nullisomic) [45]. For instance, Nulli1A-Tetra1D (N1AT1D) lacks chromosome 1A (nullisomic-1A), but contains two chromosome pairs of chromosome 1D (tetrasomic-1D), resulting a line containing no copies of chromosome 1A, two doses of chromosome 1B, and four doses of chromosome 1D. PCR analysis of genomic DNA from nulli-tetrasomic lines allows one to determine which chromosome a gene is located on based on failure to amplify in specific nullisomic lines [16]. For example, if a gene is located on chromosome 1A, the PCR product will not be generated in the N1AT1D or N1AT1B template when 1A genome-specific primers are used. PCR amplification was performed as described above and repeated three times for each primer pair.

### Quantitative Reverse Transcription-PCR (RT-qPCR) analysis

One microliter of the cDNA reaction was used as a template in the RT-qPCR reaction. Real-time quantitative PCR was performed using the LightCycler FastStart DNA Master SYBR Green I Kit on a LightCycler carousel-based system (Roche). The cDNA synthesized from six biological replicates was used to perform in RT-qPCR with two technical replications for each sample of each primer pair. The constitutive transcript of the protein transport gene *Sec23A* (*TaSEC*) was used for normalization to compensate for small variations in the input RNA amounts and cDNA synthesis efficiency among samples [46]. The expression levels of *TaEPSPS-7A1*, *TaEPSPS-7D1*, and *TaEPSPS-4A1* in hexaploid wheat were determined by genome-specific primers (Additional file 1). Each PCR reaction consisted of 1 μl of template, 0.5 μM primer pair, 3 mM MgCl_2_, but 2 mM for *TaEPSPS-7D1*, and 1X FastStart DNA Master SYBR Green I reagent (Roche) in a total volume of 10 μl. The amplification program was 95 °C for 10 min followed by 50 cycles of 95 °C for 10 s, 5 s at the specific annealing temperature (Additional file 1), and 72 °C for 10 s. After amplification, amplicon melting profiles were generated ranging over 70−96 °C to assess PCR specificity. A 2-fold serial dilution of genomic DNA or cDNA samples was used to construct a standard curve to determine amplification efficiency (99.06−106.02 %). Relative fold-change of *EPSPS* was determined using the comparative Cq or 2^-ΔΔCq^ method [47], where Cq = Cq(*TaEPSPS*) - Cq(*TaSEC*) and Cq = Cq(*TaEPSPS-7A1* or -*7D1*) - Cq (*TaEPSPS-4A1* used as a calibrator for comparisons). Total RNA without reverse transcription (no RT) was used as a negative control to screen for gDNA contamination. No template controls were included for each real-time PCR run to screen for contamination of reagents.

### Data analysis

Sequence alignments were generated using ClustalW (http://www.genome.jp/tools/clustalw/) and Geneious™ software. Amino acid sequences were deduced using ExPaSy (http://web.expasy.org/translate/.). For RT-qPCR, the mean values of *EPSPS* fold change of each genomic copy were compared by statistical analysis using a one-way general analysis of variance (ANOVA) algorithm with Tukey’s comparison at the significance level of 0.01 (SAS version 9.3). Phylogenetic trees were constructed under the genetic distance model of Tamura-Nei, where numbers indicate the genetic distance [48]. The cladograms were generated using Geneious™ software based on analysis with the UPGMA (rooted) algorithm for phylogeny for Fig. 6 or with the Neighbor-Joining (unrooted) algorithm with the bootstrap test (1000 replicates) for Fig. 7 [49, 50].

## Availability of supporting data

The data sets supporting the results of this article are included within the article and its additional files.

## Additional files

Additional file 1:
**Combinations of primer pairs for particular purposes and conditions for PCR amplification.** (PDF 81 kb) Additional file 2:
**A nucleotide sequence alignment of** ***EPSPS*** **cDNA clones from allohexaploid wheat and wheat progenitors.** (PDF 312 kb) Additional file 3:
**Nucleotide sequence alignment and primer positions.** (PDF 169 kb) Additional file 4:
**The** ***EPSPS*** **genomic DNA cloning strategy.** (PDF 245 kb) Additional file 5:
**Amino acid sequence alignment of wheat EPSPS.** (PDF 47 kb) Additional file 6:
**Conservation of the predicted signal peptide cleavage site.** (PDF 69 kb)
