# Supplementary material for: Molecular and phylogenetic characterization of the homoeologous EPSP Synthase genes of allohexaploid wheat, Triticum aestivum (L.)
Source: BMC Genomics. 2015 Oct 23;16:844. doi: 10.1186/s12864-015-2084-1 (PMC4619226; doi:10.1186/s12864-015-2084-1)
Supplement: Additional file 1: — Combinations of primer pairs for particular purposes and conditions for PCR amplification. (PDF 81 kb) [file 12864_2015_2084_MOESM1_ESM.pdf]

Additional file 1. Combinations of primer pairs for particular purposes and conditions for PCR amplification.

Primer pairs	Template	Genome	Ta(˚C)	Extension time	Amplicon size (bp)	Uses
For preliminary testing						Finding PCR condition for cDNA cloning
F2-R2	cDNA	-	50-61	1 min	~550	all but for *Ae. speltoides*
F2-R3	cDNA	-	50-61	1 min	~550	all but for *Ae. speltoides*
F2-R1	cDNA	-	50-61	1.5 min	1190	all but for *Ae. speltoides*
F3-R2	cDNA	-	50-61	1 min	~550	all wheat genotypes
F3-R3	cDNA	-	50-61	1 min	~550	all wheat genotypes
F3-R1 (selected)	cDNA	-	50-61	1.5 min	1190	all wheat genotypes
For cloning						
**Common sequence**						cDNA cloning from all wheat genotypes
F3-R1	cDNA	common	56.9-59.2	1.5 min	1190	
TaEPSPS-7A1						Cloning of full-length *TaEPSPS-7A1*
F1.2-R1	gDNA	7A	58.4	3 min	3342	
TaEPSPS-7D1						Cloning of *TaEPSPS-7D1*
F3-R2	gDNA	7D	57.3-60.5	1 min	838	normal PCR
F14-D-R1	gDNA	7D/Uk[a]	54.9	2 min	600/838[a]	normal PCR
F19-D-R18-D	gDNA	-	53	5 min	-	IPCR on *Acc* I-digested gDNA
F18-D-R18-D	gDNA	7D	53	3 min	1261	nested-IPCR on *Acc* I-digested gDNA
InvF1-AB/D-InvR1-AB/D	gDNA	-	55	5 min	-	IPCR on *Hind* III-digested gDNA
InvF3-AB/D-InvR2-AB/D	gDNA	7D	53	3 min	702	nested-IPCR on *Hind* III-digested gDNA
Int1_F2-D-R1	gDNA	7D	59	3 min	2554	normal PCR
Int3_F1-D-R1	gDNA	7D	54	2 min	1868	normal PCR
TaEPSPS-4A1						Cloning of *TaEPSPS-4A1*
F13-AB-R1	gDNA	4A	54.9	2 min	647	normal PCR
InvF1-AB/D-InvR1-AB/D	gDNA	-	55	5 min	-	IPCR on *Acc* I-digested gDNA
InvF3-AB/D-InvR2-AB/D	gDNA	4A	53	3 min	682	nested-IPCR on *Acc* I-digested gDNA
Int1_F2-A/B-R1	gDNA	4A	55	3 min	2597	normal PCR
Int3_F1-A/B-R1	gDNA	4A	54	2 min	1918	normal PCR
F3-Ex6_B-R1	cDNA	common	56.6	1 min	874	normal PCR
Ex6_B-F-R1	cDNA	4A	56.6	1 min	470	normal PCR
For mapping gene locations						Mapping chromosome location of *EPSPS* genes
F18-AB-R16-AB	gDNA[b]	7A	63	40 sec	112	
Int3_F1-AB-Ex4_R2	gDNA[b]	7A	56.6	40 sec	288	
F18-D-R16-D	gDNA[b]	7D	63	40 sec	112	
Ex6_B-F-Ex6_B-R1	gDNA[b]	4A	58	40 sec	154	
Int3_F1-A/B-Ex4_R3	gDNA[b]	4A	56.6	40 sec	338	
For determining gene expression						Measuring *EPSPS* transcription levels using RT-qPCR
F18-AB-R16-AB	cDNA	7A	63	10 sec	112	
F18-D-R16-D	cDNA	7D	63	10 sec	112	
Ex6_B-F-Ex6_B-R1	cDNA	4A	60	10 sec	154	
TaSEC_F-TaSEC_R	cDNA	-	60	10 sec	224	

[a]Uk stands for unknown PCR product named as TaUnknown (838-bp amplicon).

[b]Genomic DNA of nulli-tetrasomic lines was used as a template in PCR amplification.
