# Supplementary material for: Molecular and phylogenetic characterization of the homoeologous EPSP Synthase genes of allohexaploid wheat, Triticum aestivum (L.)
Source: BMC Genomics. 2015 Oct 23;16:844. doi: 10.1186/s12864-015-2084-1 (PMC4619226; doi:10.1186/s12864-015-2084-1)
Supplement: Additional file 2: — A nucleotide sequence alignment of EPSPS cDNA clones from allohexaploid wheat and wheat progenitors. (PDF 312 kb) [file 12864_2015_2084_MOESM2_ESM.pdf]

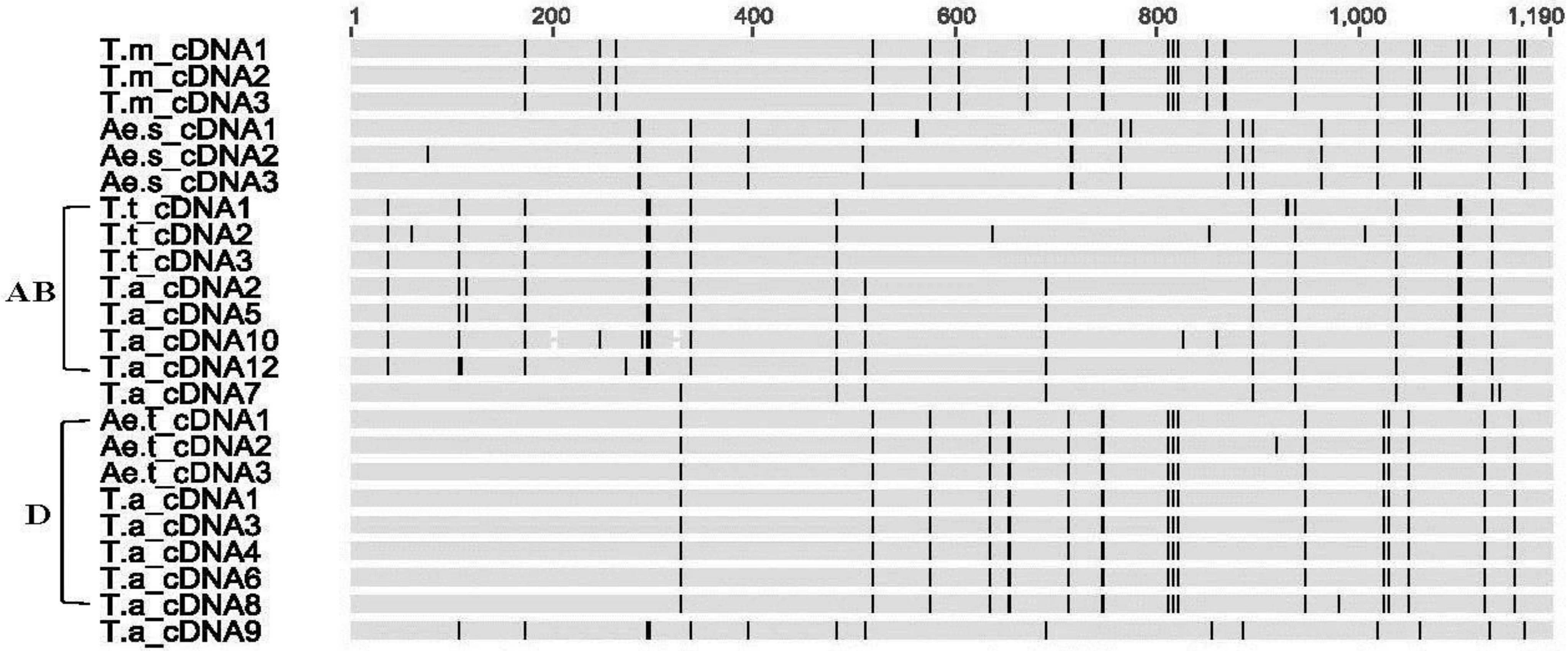

Additional file 2. A nucleotide sequence alignment of *EPSPS* cDNA clones from allohexaploid wheat and wheat progenitors. The diagram indicates the position of nucleotide polymorphisms identified in a ClustalW alignment of the 1190-bp cDNA clones of *T. aestivum* 'Louise' (T.a_cDNA#), *T. turgidum* (AB progenitor) (T.t_cDNA#), *Ae. tauschii* (D progenitor) (Ae.t_cDNA#), *Ae. speltoides* (B relative) (Ae.s_cDNA#), and *T. monococcum* (A relative) (T.m_cDNA#). Bars indicate point mutations, and dashes indicate gaps. Brackets mark the two distinct clusters of *EPSPS* cDNA sequences from allohexaploid wheat as '*EPSPS-AB'* (AB = *TaEPSPS-7A1*) and '*EPSPS-D'* (D = *TaEPSPS-7D1*) based on sequence homology to the wheat progenitors *T. turgidum* and *Ae. tauschii*, respectively.
