# Supplementary material for: Molecular and phylogenetic characterization of the homoeologous EPSP Synthase genes of allohexaploid wheat, Triticum aestivum (L.)
Source: BMC Genomics. 2015 Oct 23;16:844. doi: 10.1186/s12864-015-2084-1 (PMC4619226; doi:10.1186/s12864-015-2084-1)
Supplement: Additional file 3: — Nucleotide sequence alignment and primer positions. (PDF 169 kb) [file 12864_2015_2084_MOESM3_ESM.pdf]

```
OsEPSPS        AAATAAAAATCCCGCCACCACGGCGCGATGGTAAAGGGGGACGCTTCTAAACGGGCCGG    60
TaEPSPS-7A1    ------------------------------------------------------------
TaEPSPS-7D1    ------------------------------------------------------------
TaEPSPS-4A1    ------------------------------------------------------------

OsEPSPS        GCACGGGACGATCGGCCCCGAACCCGGCCCATCTAACCGCTGTAGGCCCACCGCCCACCA   120
TaEPSPS-7A1    ------------------------------------------------------------
TaEPSPS-7D1    ------------------------------------------------------------
TaEPSPS-4A1    ------------------------------------------------------------

OsEPSPS        ATCCAACTCCGTACTACGTGAAGCGCTGGATCCGCAACCCGTTAAGCAGTCCACACGACT   180
TaEPSPS-7A1    ------------------------------------------------------------
TaEPSPS-7D1    ------------------------------------------------------------
TaEPSPS-4A1    ------------------------------------------------------------

OsEPSPS        CGACTCGACTCGCGCACTCGCCGTGGTAGGTGGCAACCCTTCTTCCTCCTCTATTTCTTC   240
TaEPSPS-7A1    ------------------------------------------------------------
TaEPSPS-7D1    ------------------------------------------------------------
TaEPSPS-4A1    ------------------------------------------------------------

OsEPSPS        TTCTTCCTCCCTTCTCCGCTCACCACACCAACCGCACCAACCCCAACCCCGCGCGCGCTC   300
TaEPSPS-7A1    ------------------------------------------------------------
TaEPSPS-7D1    ------------------------------------------------------------
TaEPSPS-4A1    ------------------------------------------------------------

OsEPSPS        TCCCCTCTCCCCTCCCACCAACCCCACCCCATCCTCCCGACCTCCACGCCGCCGGCAATG   360
TaEPSPS-7A1    ------------------------------------------------------------
TaEPSPS-7D1    ------------------------------------------------------------
TaEPSPS-4A1    ------------------------------------------------------------
                         +1
OsEPSPS        GCGGCGACCATGGCGTCCAACGCCGCGGCTGCGGCGGCGGTGTCCCTGGACCAGGCCGTG   420
TaEPSPS-7A1    ------GCAATGGCGATGGCTGCCGCGGCGACCGTGGCCGCGTCCGCGTCCTCCAGCGCG    54
TaEPSPS-7D1    ------------------------------------------------------------
TaEPSPS-4A1    ------------------------------------------------------------
                     F1.2 →

OsEPSPS        GCGGCGTCGGCGGCGTTCTCGTCGCGGAAGCAGCTGCGGCTGCCCGCCGCGGCGCGCGGG   480
TaEPSPS-7A1    GTGTCGCTCGACCGCGCCGCCCCGGCCCACCCGCGCCGGCTGCGGATGCCGGCGGCCCGC   114
TaEPSPS-7D1    ------------------------------------------------------------
TaEPSPS-4A1    ------------------------------------------------------------

OsEPSPS        GGGATGCGGGTGCGGGTGCGGGCGCGGGGGCGGCGGCGGGAGGCGGTGGTGGTGGCGTCCGCG   540
TaEPSPS-7A1    GCGGCCCAC---CGCGGGGCGGTGCGGCTGTGGGGGCCCCGCGGCGCGGCGGCGCGCGCG   171
TaEPSPS-7D1    ------------------------------------------------------------
TaEPSPS-4A1    ------------------------------------------------------------

OsEPSPS        TCGTCGTCGTCGGTGGCAGCGCCGGCGGCGAAGGCGGAGGAGATCGTGCTCCAGCCCATC   600
TaEPSPS-7A1    ACGTCCGTGGCGGCCCCCGCGGCGCCCGCGGGCGCCGAGGAGGTCGTGCTGCAGCCCATC   231
TaEPSPS-7D1    ------------------------------------------------------------
TaEPSPS-4A1    ------------------------------------------------------------

OsEPSPS        AGGGAGATCTCCGGGGCGGTTCAGCTGCCAGGGTCCAAGTCGCTCTCCAACAGGATCCTC   660
TaEPSPS-7A1    CGCGAGATCTCCGGCGCCGTGCAGCTGCCCGGCTCCAAGTCGCTCTCCAACCGGATCCTC   291
TaEPSPS-7D1    ------------------------------------------------------------
TaEPSPS-4A1    ------------------------------------------------------------

OsEPSPS        CTCCTCTCCGCCCTCTCCGAGGTGAGACGCGGATCCCTTCCTCTTGCGTGAATTCCATTT   720
TaEPSPS-7A1    CTCCTCTCCGCCCTCTCCGAGGTGA--------CTCCCCCCTCCTCCCGCTCTTCCTTTG   343
TaEPSPS-7D1    ------------------------------------------------------------
TaEPSPS-4A1    ------------------------------------------------------------
```

```
OsEPSPS        CTGGAGATGAGATTTTAGGGGGTTTATTAGGTGAGGTGGCTGTGTTTGTGAAATCCTAGG    780
TaEPSPS-7A1    GGTGATGCGAATTGGGTGCAGAGATGGGATTCTAGGGGGTTTAGCTGACGCACCCTGTCA    403
TaEPSPS-7D1    ------------------------------------------------------------
TaEPSPS-4A1    ------------------------------------------------------------

OsEPSPS        AATTATCTCTCAAGTCAATCTAACGATGAGATATAACTGAGGTTCTGGTTTTAATCACAC    840
TaEPSPS-7A1    TGCTAGCT---------AGGTGCTCGCGAGATCATGGGGATTTTAACTCCCCGACCATAG    454
TaEPSPS-7D1    ------------------------------------------------------------
TaEPSPS-4A1    ------------------------------------------------------------

OsEPSPS        ACTCATATAACCAATTTATTGAAACATTTTGGTTTGGCATAAGAAACTGCTTACGAAGGT    900
TaEPSPS-7A1    GTTAACGAGGGCAGGTTGTT-------TGGTAGCCAATTCATGCGTTTGCTTACCAGATC    507
TaEPSPS-7D1    ------------------------------------------------------------
TaEPSPS-4A1    ------------------------------------------------------------

OsEPSPS        ATGATATCCTCCTACATGTCAGGCTACTAAATTTTCACGACGGTATGATCCACTCAAAAC    960
TaEPSPS-7A1    ATTGTATTGTGTAGCTTGTATTTGGATCAAGATATCGATGCATCAGAATGACCAGGAGAC    567
TaEPSPS-7D1    ------------------------------------------------------------
TaEPSPS-4A1    ------------------------------------------------------------

OsEPSPS        AAGTTTCTTAACGAGTCTGGTGAGGTCTGTTATGAAATTTGTGTAAACTAAGGCAACTTT   1020
TaEPSPS-7A1    CCTCCTCTAATTTAGGCTACCAAATTGTTCTGATGGCTCCTTTATGAAAAGTAGCATGTT    627
TaEPSPS-7D1    ------------------------------------------------------------
TaEPSPS-4A1    ------------------------------------------------------------

OsEPSPS        GGAGGTTTCGCACTGTACCAATGTTATGTTTGAACATTTTGCAAGCAGTGCTTTCTCCCA   1080
TaEPSPS-7A1    GTAATGCTGTTATGAACTCTTATTCATGTATCTGCATTTAAAATGGTGCAACCTAAGGTC    687
TaEPSPS-7D1    ------------------------------------------------------------
TaEPSPS-4A1    ------------------------------------------------------------

OsEPSPS        AAATTATGCAATTTTGAGGCTCCTCTACATCATTATAATTCCCCAATACATTGCTCTTTA   1140
TaEPSPS-7A1    TATTCAC---ATTTTGTGAGACGGTGTGACCAC--AAAAGCCG----ATACTATTTTACA    738
TaEPSPS-7D1    ----CAC---ATTCTGTGAGACGACGTCATCATGAAAAAGGCG----ATATTATTTTTGA     49
TaEPSPS-4A1    ----CAC---ATTTTGTGAGACGGCGTGGCCACTAAAAGACAG----ATATTATTTTAGA     49

OsEPSPS        TTCTTAATAGCTTTGATCGCGAAATTTAACATTTTAATTCTTGAGCTGTTATTTTGTAGC   1200
TaEPSPS-7A1    CGAGAAATC-GTTGAATTGCTATTCGTGGCATTAGTTTTCATGAGCTATATAGGTACTAA    797
TaEPSPS-7D1    TGAGAAATC-CTTGAATTGCTATTTGTAGCATTTGTTTTCATGAGCTATATAGGTACTAA    108
TaEPSPS-4A1    TGAGAAATC-CTTGAATTGCTATTCG-ACTATT---------------------------     80
                        ----------------------->
                             Int1_F2-A/B

OsEPSPS        ATCAGTTTATCATGAGCCATGTTTGGTACTAAATATACAATCCCTTGGGTTTATTTGTTT   1260
TaEPSPS-7A1    ATC----TAGGATCCTCCAGGCTTTATAT-GTTTCCACTGATCAGAAAGCAATATTGCAG    852
TaEPSPS-7D1    ATCAAGGTAGGATCCTTAAGGCTTTATATCGTTTCCACTGATCAGAAAACTATAGAGCAG    168
TaEPSPS-4A1    -----GGTAGGATCCTCCACGCTTTATATGGTTTCCACTGATCAGAAAACAATATTGCAG    135
                                                  ----------------------->
                                                       Int1_F2-D

OsEPSPS        CCAAGCATGTCAT-TAACTTATCTTAATGTGGACAAGAAACTGATGCCTGCTTACATTGC   1319
TaEPSPS-7A1    CATGTCCTGTTCTCTTACTTATTTATAGTAAGATGAGAAATTGGAGCTGGTTTACTTCAC    912
TaEPSPS-7D1    CATGTCCTGTTATCTTATTTATTTATAGTAAGATGAGAAATTGGAGCTGGTTTACTTCAT    228
TaEPSPS-4A1    CATGTCCTGTTCTCTTGCTTATTTATAGTAAGATGAGAAATTGGAGCTGGTTTACTTCAC    195

OsEPSPS        TATTATTTCAAGCGGGTATTGATCCTTTGACATGTGATTGATCATTTTTTTTTCTCTGGT   1379
TaEPSPS-7A1    TGTCATCTCCAGC------TGATCCGTAG--------------------CTTGTTTCTGGT    947
TaEPSPS-7D1    TGTCATCTCTAGC------TGATCCGTAG--------------------CTTGTCTCTGCT    263
TaEPSPS-4A1    TGTCATATCTAGC------TGATCTGTAG--------------------CTTGTTTCTGCT    230
```

```
OsEPSPS        TATTAGGGCACAACAGTGGTGGACAACTTGCTGAACAGTGAGGATGTTCACTACATGCTT   1439
TaEPSPS-7A1    CATTAGGGAACAACGGTGGTGGATAACCTGTTGAACAGTGAGGATGTCCACTACATGCTT   1007
TaEPSPS-7D1    CATTAGGGAACAACGGTGGTGGATAACCTGTTGAACAGCGAGGATGTCCACTACATGCTC    323
TaEPSPS-4A1    CATTAGGGAACAACGGTGGTGGATAACCTGTTGAACAGCGAGGATGTCCACTACATGCTC    290
                ******* ***** ******** *** ** ******* ******** ***********
                                                        ------------->
                                                            F2, F3

OsEPSPS        GAGGCCCTGAAAGCCCTCGGGCTCTCTGTGGAAGCAGATAAAGTTGCAAAAAGAGCTGTA   1499
TaEPSPS-7A1    GAGGCCCTGGAAGCCCTTGGACTCTCCGTGGAAGCAGATAAAGTTGCAAAAAGAGCTGTG   1067
TaEPSPS-7D1    GAGGCCCTGGAAGCCCTCGGACTCTCCGTGGAAGCAGATAAAGTTGCAAAAAGAGCTGTG    383
TaEPSPS-4A1    GAGGCCCTGGAAGCCCTTGGACTCTCCGTGGAAGCAGATAAAGTTGCAAAAAGAGCTGTG    350
               ********* ******* ** ***** ********************************

OsEPSPS        GTCGTTGGCTGTGGTGGCAAGTTTCCTGTTGAGAAGGATGCGAAAGAGGAAGTGCAACTC   1559
TaEPSPS-7A1    GTTGTTGGCTGTGGCGGCAGGTTCCCAGTCGAAAAGGACGCCAAAGAGGAAGTAAAGCTC   1127
TaEPSPS-7D1    GTTGTTGGCTGTGGCGGCAGGTTCCCGGTCGAAAAGGACGCCAAAGAGGAAGTAAAGCTC    443
TaEPSPS-4A1    GTTGTTGGCTGTGGCGGCAGGTTCCCGGTCGAAAAGGACGCCAAAGAGGAAGTAAAGCTC    410
               ** *********** **** *** ** ** ** ***** ** ***********  * ***

OsEPSPS        TTCTTGGGGAACGCTGGAACTGCAATGCGACCATTGACAGCAGCCGTGACTGCTGCTGGT   1619
TaEPSPS-7A1    TTCTTGGGTAATGCTGGAACTGCAATGCGGCCACTGACGGCAGCTGTAGTAGCTGCTGGT   1187
TaEPSPS-7D1    TTCTTGGGTAATGCTGGAACTGCAATGCGTCCATTGACGGCAGCTGTAGTAGCTGCTGGT    503
TaEPSPS-4A1    TTCTTGGGTAATGCTGGAACTGCAATGCGGCCATTGACGGCAGCTGTAGTTGCTGCTGGT    470
               ******** ** ***************** *** **** ***** **    *********

OsEPSPS        GGAAATGCAACGTATGTTTTTTTTTTTAATGTTTATGAAAATATGTATGGAATTCATGGG   1679
TaEPSPS-7A1    GGAAATGCAACGTATGTTTTCTTTATCCTAGTGGAAATAAGTATGAGATCCATGGGTATG   1247
TaEPSPS-7D1    GGAAATGCAACGTATGTTTTCTTTATCCTAGTTGAAATAAGTATGAGATCCATGGGTATG    563
TaEPSPS-4A1    GGAAATGCAACGTATGTTTTCTTTATCCTAGT-GAAATAAGTATGAGATCCATGGGTATG    529
               ***********

OsEPSPS        GTATGTTTTATGACCTTTTTCTTTAC-----------CATCAGTTATGTGCTTGATGGAG   1728
TaEPSPS-7A1    TTTGGAGACTGATCGTTTCTTTTATT--AAAAAAAAACTTCAGTTATGTGCTTGATGGCG   1305
TaEPSPS-7D1    TTTGGAGACTGATCGTGTCTTTTATT-AAAAAAAAAACTTCAGTTATGTGCTTGATGGAG    622
TaEPSPS-4A1    TTTGGAGACTGATCATGTCTTTTATTTAAAAAAAAAACTTCAGTTATGTGCTTGATGGAG    589
                                                          *************** *

OsEPSPS        TGCCACGAATGAGGGAGAGACCGATTGGTGACTTGGTTGTCGGGTTGAAACAACTTGGTG   1788
TaEPSPS-7A1    TACCAAGAATGAGGGAGCGACCTATTGGTGACTTAGTTGTAGGTTTGCAACAACTCGGCG   1365
TaEPSPS-7D1    TACCAAGAATGAGGGAGCGACCTATTGGTGACTTAGTTGTAGGTTTGCAACAACTCGGCG    682
TaEPSPS-4A1    TACCAAGAATGAGGGAGCGACCTATTGGTGACTTGGTTGTCGGTTTGCAACAACTCGGCG    649
               * *** *********** **** *********** ***** ** *** ******* ** *

OsEPSPS        CGGATGTCGACTGTTTCCTTGGCACTGAATGCCCACCTGTTCGTGTCAAGGGAATTGGAG   1848
TaEPSPS-7A1    CAGATGTCGATTGTTTCCTTGGCACAAACTGCCCACCTGTCCGTATCAACGGCAAAGGAG   1425
TaEPSPS-7D1    CAGATGCTGATTGTTTCCTTGGCACTAACTGCCCACCTGTTCGTATCAATGGCAAAGGAG    742
TaEPSPS-4A1    CAGATGCTGATTGTTTCCTTGGCACTAACTGCCCACCTGTCCGTATCAATGGCAAAGGAG    709
               * ****  ** **************  * *********** *** **** ** *  ****

OsEPSPS        GACTTCCTGGTGGCAAGGTTAGTTACTCCTAAACTGCATCCTTTGTACTTCTGTATGCAC   1908
TaEPSPS-7A1    GTCTACCTGGTGGCAAGGTTAGCTACTCATCAACTTGCATGTTATCTACTTT-GTGCACA   1484
TaEPSPS-7D1    GGCTACCTGGTGGCAAGGTTAGCTACTTGTGAACTTGCATGTTATGTACTTTTGTGCACA    802
TaEPSPS-4A1    GGCTACCTGGTGGCAAGGTTAGCTACTCGTGAAATTGCATGTTATGTACTTTTGAGCACA    769
               * ** ****************

OsEPSPS        CTCAATTCTTTGTCAACCTTCTGCATTTATAAGGAACATTCTATGATGCAATTCGACCTT   1968
TaEPSPS-7A1    C-----------------------------------------------------------   1485
TaEPSPS-7D1    ------------------------------------------------------------
TaEPSPS-4A1    ------------------------------------------------------------

OsEPSPS        ACACTGCACAGTAACTTGAAATGTTTCATGCTTAATCAATATGCCATATTCCTGCCAAGC   2028
TaEPSPS-7A1    --------TTTTGTTCTCTGTCAGAGAAGT----TATTATTATGGCTAGTACGATGTAAT   1533
TaEPSPS-7D1    --------TTCAGTTCTCTGTCAAAGAAAAATAATATTATTATGGGTAGTACGATGCAAC    854
TaEPSPS-4A1    --------TTCAGTTCTCTGTCAAAGAAAACAATATTAT-ATTGGTAGTACGAT-CAAT    819
```

```
OsEPSPS         TCAAGCGAGCAATATTTGTTTGAATTTGGTACCATATTTTTGTATATTTGGGCATTCCTT   2088
TaEPSPS-7A1     GCAACCTTAGACCGCGCACTAAATTTTAAGAACCAATCAACATTTAATAATTTCAATATG   1593
TaEPSPS-7D1     GCGACCTTACACCGTGCACTAAAGTTTAAGAACCAATAAACATTGAATAAATTCAATACT    914
TaEPSPS-4A1     GCGACCTTACACAATGCACTAAAGTTGAAGAACCAATAAACATTGAATAATTTCAATACT    879
```

Int3_F1-AB, -A/B, -D

```
OsEPSPS         TTTGGTCTTGATGTCTTCTTTTGAATTAGCATTTAACTGAATTACACTCAACAGGTTAAG   2148
TaEPSPS-7A1     TGAGTATTGCATGATCTATGATAGCTTGGTGCTAAAGATTGAATGATTTCACAGGTTAAG   1653
TaEPSPS-7D1     TGAGTATTGCGTGATCAATCATAGATTGGCGCTTAACATTGAATAATTTCACAGGTTAAG    974
TaEPSPS-4A1     TGAGTATTGCATGATCAATCATAGATTGACACTTAATATTGAATAATTTCACAGGTTAAG    939
                                                               *  **********

OsEPSPS         CTCTCTGGTTCCATCAGCAGTCAGTACTTGAGTGCCTTGCTGATGGCTGCTCCTTTGGCC   2208
TaEPSPS-7A1     CTCTCTGGTTCCATTAGCAGTCAATACCTGAGTTCCTTGCTGATGGCTGCTCCTTTGGCT   1713
TaEPSPS-7D1     CTCTCTGGTTCCATTAGCAGTCAATACCTGAGTTCCTTGCTGATGGCTGCTCCTTTGGCT   1034
TaEPSPS-4A1     CTCTCTGGTTCCATTAGCAGTCAATACCTGAGTTCCTTGCTGATGGCTGCTCCTTTGGCT   999
                ************** ******** *** ***** *************************

OsEPSPS         CTTGGGGATGTGGAGATCGAAATCATTGACAAACTAATCTCCATTCCTTACGTTGAAATG   2268
TaEPSPS-7A1     CTTGAGGATGTCGAGATTGAAATCATTGATAAACTGATCTCCGTTCCTTATGTTGAAATG   1773
TaEPSPS-7D1     CTTGAGGATGTCGAGATTGAAATCATTGATAAACTGATCTCCGTTCCTTACGTTGAAATG   1094
TaEPSPS-4A1     CTTGAAGATGTCGAGATTGAAATCATTGATAAACTGATCTCCGTTCCTTACGTTGAAATG   1059
                ****  ***** ***** *********** ***** ****** ******* *********

OsEPSPS         ACATTGAGATTGATGGAGCGTTTTGGTGTGAAGGCAGAGCATTCTGATAGTTGGGACAGA   2328
TaEPSPS-7A1     ACATTGAAATTGATGGAGCATTTTGGTGTGACTGCGGAGCATTCTGATAGTTGGGACAGA   1833
TaEPSPS-7D1     ACATTGAAATTGATGGAGCGTTTTGGCGTGACTGCGGAGCATTCTGATAGTTGGGACAGA   1154
TaEPSPS-4A1     ACATTGAAATTGATGGAGCGTTTTGGCGTGACTGCGGCGCATTCTGATAGTTGGGACAGA   1119
                ******* *********** ****** ****  ** * **********************

OsEPSPS         TTCTATATTAAGGGAGGGCAGAAGTACAAGTAAGCTTCTACCT-----------------   2371
TaEPSPS-7A1     TTCTACATTAAGGGAGGACAAAAATACAAGTAAGTTCTACATTTTCCTACTTCTTCTAAT   1893
TaEPSPS-7D1     TTCTACATTAAGGGAGGACAAAAGTACAAGTAAGTTCTACATT-----------------   1197
TaEPSPS-4A1     TTCTACATCAAGGGAGGACAAAAATACAAGTAAGTTCTACATT-----------------   1162
                ***** ** ******** ** ** *****

OsEPSPS         ------------------------------------------------------------
TaEPSPS-7A1     AAACTTTTAATTGTTTCATTTCTTCTAATAGTGGAGTACTAATGAAAGATATCAGTTTTC   1953
TaEPSPS-7D1     ------------GCTTTACTTCTTCTGATAGTGGAGTACAAAAGCATGATTCTAGTTTTC   1245
TaEPSPS-4A1     ------------GCTTTACTTCTTCTGATAGTGGAGTACAAAAGCATGATTCTAATTTTC   1210

OsEPSPS         ------------------------------------------------------------
TaEPSPS-7A1     AGTTTGCC-AAATATTTACAGACGCAACCCCATT-GAATTATGGGTAAACTAGGAAATAA   2011
TaEPSPS-7D1     AGTCTACCCAAATATTTATAGACGCACCCCCATTTGAATTATTGATAAACTAGGAAATAA   1305
TaEPSPS-4A1     TGTCTACCCACATATTTATAGACGCACCCCCATTTGAATTATTGATAAACTAGGAAATAA   1270

OsEPSPS         ------------------------------------------------------------
TaEPSPS-7A1     ATGTTTTACTAAAACTATTGACTGCTCAGTAGCTATGAAGGTGCACTGTACTATGAAACC   2071
TaEPSPS-7D1     ATATTGTACAAAATCTGTCGACTGCTCAGTAGCTTTCAAGGCGCACTGTACTAGGAAACC   1365
TaEPSPS-4A1     ATATTGTACAAAATCTGTCGACTGCTCAGTAGCTCTGAAGGCGCACTGTGCTAGGAAACC   1330

OsEPSPS         ------------------------------------------------------------
TaEPSPS-7A1     AGTGCCTACTGCCTAGGCAATCGGCCCCACTGCTTACAATGAATTGTACAGTTACGTTTT   2131
TaEPSPS-7D1     AGTGCCTACTGCCTAGGCAATCGACCCTGCTACCTACAATGCATTGCACAGTTCTGTTTT   1425
TaEPSPS-4A1     AGTGTCTACTGCCTAGGCAATCAACCCTTCTACCTACAATGCATTGCACAGTTCTGTTTT   1390

OsEPSPS         ------------------------------------------------------------
TaEPSPS-7A1     CTGGTACATGGACTTGATCACACCAGAAGTATAATCCATCTTAAAAATGGAATGAAAATA   2191
TaEPSPS-7D1     CTGG-ACATAGACTTGATCACACCAGAAGTATCATCCATCTTAAAATTGGACTGAAAATA   1484
TaEPSPS-4A1     CTGG-ACATATACTTGATCACACCAGAGGTATCATCCATCTTAAAATTGGACTGAAAATA   1449
```

```
OsEPSPS         --------------------------------GCCTTACTGAGCTGAATTATTCGGGTGT   2399
TaEPSPS-7A1     GAGAATAT----------TTTGCGATGAGCATCTATGCAGATGATTCTTGATTTTGCTTC   2241
TaEPSPS-7D1     GAGGATATCGGCAGTTGTTTGGCAATGAGCATCTATGCAGATGATACTTGATTTTGCTTC   1544
TaEPSPS-4A1     GAGAACATCGGCAGTTGTTTGGCAATGAGCATCTATGCAGATGATTCTTGATTTTGCTTC   1509

OsEPSPS         TTATGATTAA--------------------------------------------------   2409
TaEPSPS-7A1     TTTTGGCCCTTTCT-AAGTTATGCCCTTGCTGCTATATGTC------------ACATCTA   2288
TaEPSPS-7D1     TTTTGACCCTTTTT-ATCTTAGGCCCTTGCTGCCATATGGCCCATCAGGATACACATCCA   1603
TaEPSPS-4A1     TTTTGGCCCTTTTTTATCTTATGCCCTTGCTGCCATATGGCCCATCAGGATACACATCCA   1569

OsEPSPS         ------------------------------------------------------------
TaEPSPS-7A1     CGCTTTTACTGAACATACTCCTCTTATGTACTCGGAAACTGTTAGTCCAAACTTCATAAA   2348
TaEPSPS-7D1     TGCTTTTACTGAACATACTC---TTATGTACTCGGAAACTGTTAGTTCAAACTTCATAAA   1660
TaEPSPS-4A1     TGCTTTTACTGAACATACTC---TTATGTACTCGGAAACTGTTAGTTTAAACTTCATAAA   1626

OsEPSPS         -----------CTCCCTAAACTAACCCTTTTTCTTTTCTTTGGCATTGACAGATCTCCTG   2458
TaEPSPS-7A1     AGTTGCAGTAACCATTTCCTAAACGAGCCATCCATTATTCTGGAATCGACAGGTCCCCTG   2408
TaEPSPS-7D1     AGTTGCAGTAACCATTTCCTAAACGAGCCATCCGTT-CTCTGGAATCGACAGGTCCCCTG   1719
TaEPSPS-4A1     AGTTGCAGTAACCATTTCCTAAACGAGCCATCTGTT-CTCTGGAATCGACAGGTCCCCTG   1685
                                                                     ** ****

OsEPSPS         GAAATGCCTATGTTGAAGGTGATGCCTCAAGCGCGAGCTATTTCTTGGCTGGTGCTGCAA   2518
TaEPSPS-7A1     GAAATGCCTATGTCGAAGGTGATGCCTCAAGTGCGAGCTATTTCTTGGCTGGTGCTGCCA   2468
TaEPSPS-7D1     GAAATGCCTATGTCGAAGGTGATGCCTCAAGTGCGAGCTATTTCCTGGCTGGTGCTGCCA   1779
TaEPSPS-4A1     GAAATGCCTATGTTGAAGGTGATGCCTCAAGTGCGAGCTATTTCCTGGCTGGTGCTGCCA   1745
                ************* ***************** ************ ************* *

OsEPSPS         TCACTGGAGGCACTGTGACAGTTCAAGGTTGTGGTACGACCAGTTTGCAGGTATAACTGT   2578
TaEPSPS-7A1     TCACCGGAGGGACTGTGACTGTCGAAGGTTGCGGCACCACTAGTTTGCAGGTAAAATTGT   2528
TaEPSPS-7D1     TCACTGGAGGGACTGTGACTGTCGAAGGTTGCGGCACCACCAGTTTGCAGGTAAAATTCT   1839
TaEPSPS-4A1     TCACCGGAGGGACTGTGACTGTCGAAGGTTGCGGCACCACCAGTTTGCAGGTAAAATTCT   1805
                **** ***** ******** **  ******* ** ** ** **********

OsEPSPS         AGTGCCTGTTTTGACATTCTACCGTTTAGTCAAGTTTAGTCAGTAGTCACATATTCAGAA   2638
TaEPSPS-7A1     ACTATCCATTTTCACCCTTTTGGTTGAGCATAC---------------------------   2561
TaEPSPS-7D1     ACTATCCATTTTTACC-TTTTGGTTGAGCATACTTGACATATTACATTATTGAATGTATA   1898
TaEPSPS-4A1     ACTATCCATTTTTACCGTTTTGGTTGAGCATACTTGACATATTACATTGTTGAATGTATA   1865

OsEPSPS         TATAGCACAATCTGTATTATGCCACTGTTAATCAAATACGCTTGACCTAGAGAGTGCTAT   2698
TaEPSPS-7A1     ----------------------------------------------TGTCTTA-------   2568
TaEPSPS-7D1     TA----------------------------CCATTTTCAACCAAACATGCTTTAAGTGGAT   1931
TaEPSPS-4A1     ------------------------------CCATTTTCAACCAAACATGCTTTAAGTGGAT   1896

OsEPSPS         ATACCCTAGCTTAATCTTCAAACTAAACAGTTCTCTTGTGGCTTGCTGTGCTGTTATGTT   2758
TaEPSPS-7A1     --------------------------------------------------TGCTGTCATT   2578
TaEPSPS-7D1     ATTCGTAGCTGAATCTCTCAAACTGCTGTATTCTGTAAGTGGCTTCCATGTGCTGTCATT   1991
TaEPSPS-4A1     ATTCGTAGCTGAATCTGTCAAACTGCTGTATTCTGTAAGTGGCTTCCATGTGCTGTCATT   1956

OsEPSPS         CCCTGACCTACATGTTAATATTACAGGGTGATGTCAAATTTGCTGAGGTACTTGAGATGA   2818
TaEPSPS-7A1     CTCCCTTACTAAGGTTAATCTTGCAGGGTGATGTGAAATTTGCTGAGGTACTTGAAATGA   2638
TaEPSPS-7D1     CTCCCTGACGATGTGTAATATTGCAGGGTGATGTGAAATTCGCTGAGGTACTTGAAATGA   2051
TaEPSPS-4A1     CTCCCTGACGATGTGTCACATTGCAGGGTGATGTGAAATTTGCTGAGGTACTTGAGATGA   2016
                                          ******** ***** ************** ****

OsEPSPS         TGGGAGCAAAGGTTACATGGACTGACACCAGTGTAACCGTAACTGGTCCACCACGTGAGC   2878
TaEPSPS-7A1     TGGGAGCAAAGGTCACATGGACTGACACTAGTGTAACTGTTACTGGCCCACCGCGTCAGC   2698
TaEPSPS-7D1     TGGGAGCTAAGGTTACATGGACTGACACTAGTGTAACTGTTACTGGCCCACCGCGTCAGC   2111
TaEPSPS-4A1     TGGGAGCGAAGGTTACATGGACTGACACTAGTGTAACTGTTACTGGCCCACCGCGTCAGC   2076
                ******* ***** ************** ******** ** ***** ***** *** ***
```

Ex6_B-F

```
OsEPSPS             CTTATGGGAAGAAACACCTGAAAGCTGTTGATGTCAACATGAACAAAATGCCTGATGTTG   2938
TaEPSPS-7A1         CATTTGGAAGGAAACACCTAAAAGCTGTTGATGTCAACATGAACAAAATGCCAGATGTCG   2758
TaEPSPS-7D1         CCTTTGGAAGGAAACACTTAAAGGCTGTCGATGTCAACATGAACAAAATGCCTGATGTCG   2171
TaEPSPS-4A1         CCTTTGGAAGGAAACACCTAAAAGCTGTTGATGTCAACATGAACAAAATGCCTGATGTCG   2136
                    * * *** * ******* * ** ***** *********************** ***** *

OsEPSPS             CCATGACCCTTGCCGTTGTTGCACTCTTCGCTGATGGTCCAACTGCTATCAGAGATGGTA   2998
TaEPSPS-7A1         CGATGACTCTAGCCGTTGTTGCCCTGTTTGCCGATGGTCCAACCGCTATCAGAGATGGTA   2818
TaEPSPS-7D1         CCATGACTCTTGCCGTTGTTGCCCTCTTTGCCGATGGTCCAACTGCTATCAGAGATGGTA   2231
TaEPSPS-4A1         CAATGACTCTTGCCGTTGTTGCCCTCTTTGCTGATGGTCCAACTGCTATCAGAGATGGTA   2196
                    * ***** ** *********** ** ** ** *********** *************
                            Ex6_B-R1

OsEPSPS             AACATTAAGGCCTATTATACCTGTTCTATCATACTAGCAATTACTGCTTAGCATTGTGAC   3058
TaEPSPS-7A1         TACGTTCTATGGCCTCCCATACCTATCATGT--------GCGTAGCGATTACTCTGTTAG   2870
TaEPSPS-7D1         AACTTTCTATGGCCTTCCATACCAAGCAATCTAGCACGTGCGTCGCGGTTACTCGGT-AG   2290
TaEPSPS-4A1         AACTTTTTATGGCCTCCCATACCTATCATGTGCGTA---GCGTAGCGATTACTCTGTTAG   2253

OsEPSPS             AAAACAAATAACCAAACTTTCTTCAAAATAACTTAGAAATATAAGAAGGGTTCGTTTTGT   3118
TaEPSPS-7A1         CATGAGGGAGAGGACAAAGGACTTGAAATCACCCTGTTATGTGTTGCTGTTGATTTTGTT   2930
TaEPSPS-7D1         CATCAGGGAGGGGATAAAGGACTCGAAATCACCCTGTTATGT------------------   2332
TaEPSPS-4A1         CATCAGGGAGGGGACAAAGGACTTGAAAGCACCCTGTTATGTGTTGCTGTTGATTT-GTT   2312

OsEPSPS             GTGGTAAACAGTACTACTGTAGTTTCAGCTATGAAGTTTGCTGCTGGCAATTTTCTGAAC   3178
TaEPSPS-7A1         TTACATGTTTTGGTCCTGACACTTGTTTTGGAATCA------------------------   2966
TaEPSPS-7D1         --------------CCTGACACTTGTTC----ATT-------------------------   2349
TaEPSPS-4A1         TTACATGTTTTGGTCCTGACACTTGTTC----ATT-------------------------   2343

OsEPSPS             GGTTTCAGCTAAATTGCATGTTTGTTCATCATACTTATCCATTGTCTTCCACAGTGGCTT   3238
TaEPSPS-7A1         -------------------------------------------CTCTTCTGCAGTTGCCT   2983
TaEPSPS-7D1         -------------------------------------------CTCTTCTGCAGTTGCCT   2366
TaEPSPS-4A1         -------------------------------------------CTCTGCTGCAGTTGCCT   2360
                                                                          * ** *

OsEPSPS             CCTGGAGAGTAAAGGAAACCGAAAGGATGGTTGCAATTCGGACCGAGCTAACAAAGGTAA   3298
TaEPSPS-7A1         CCTGGAGAGTGAAGGAAACTGAAAGAATGGTCGCGATCCGGACCGAGCTGACGAAGGTAA   3043
TaEPSPS-7D1         CCTGGAGAGTGAAGGAAACCGAAAGAATGGTTGCGATCCGGACCGAGCTGACGAAGGTAA   2426
TaEPSPS-4A1         CCTGGAGAGTGAAGGAAACCGAAAGAATGGTCGCGATCCGGACCGAGCTGACGAAGGTAA   2420
                    ********** ******** ***** ***** ** ** *********** ** ***

OsEPSPS             ATTCATTAGGTCCCGTGTCCTTTCATTCTTCAAGTAGTTTGTTCATAAGTTGAATTCTCC   3358
TaEPSPS-7A1         TAATACACA-TGTCTCTTGTTCTTATATCTCAGCCTCCTGTTGCACCCCATG--------   3094
TaEPSPS-7D1         TAATACACAATGTCTCCTGTTCTTATATCTCAGCTTCCTG--------------------   2466
TaEPSPS-4A1         TAAAACACAATGTCTCCTGTTCTTATACCCCAGCCTCCTGTTGCACCCCTCGGCACTTC-   2479

OsEPSPS             TTCAATGATGTTTAAATTCATCATCTTCTTTTTTGGTGTTGTGCCAGCTGGGAGCATCGG   3418
TaEPSPS-7A1         ---------------TGCTTACCTCTCCTGTGTCCTGTGCCCTATAGCTGGGAGCAACGG   3139
TaEPSPS-7D1         ---------------ATCTAACCTGTCCTGTGCCCTGTGCCCTATAGCTGGGAGCAACGG   2511
TaEPSPS-4A1         ---------------ATCTGACCTGTCCTTTGCA-TGTGCCCTATAGCTGGGAGCAACGG   2523
                                                                   ********* ***

OsEPSPS             TTGAAGAAGGTCCTGACTACTGCATCATCACCCCACCGGAGAAGCTGAACATCACGGCAA   3478
TaEPSPS-7A1         TGGAGGAAGGCCCGGACTACTGCATCATCACGCCGCCGGAGAAGCTGAACATCACGGCGA   3199
TaEPSPS-7D1         TGGAGGAAGGCCCGGACTACTGCATCATCACGCCGCCAGAGAAACTGAACGTCACGGCGA   2571
TaEPSPS-4A1         TGGAGGAAGGCCCGGACTACTGCATCATCACGCCACCGGAGAAGCTGAACGTCACGGCGA   2583
                    * ** ***** ** ***************** ** ** ***** ****** ******* *

OsEPSPS             TCGACACCTACGATGATCACAGGATGGCCATGGCCTTCTCCCTCGCTGCCTGCGCCGACG   3538
TaEPSPS-7A1         TCGACACCTACGATGACCACCGGATGGCGATGGCCTTCTCCCTGGCGGCCTGTGCTGAGG   3259
TaEPSPS-7D1         TCGATACCTACGATGACCACCGGATGGCGATGGCCTTCTCCCTGGCGGCCTGCGCCGAGG   2631
TaEPSPS-4A1         TCGACACCTACGACGACCACCGGATGGCGATGGCCTTCTCCCTGGCGGCCTGCGCCGAGG   2643
                    **** ******** ** *** ******* ************** ** ***** ** ** *
                F18-D, F18-AB
```

```
OsEPSPS        TGCCCGTGACGATCAGGGACCCTGGTTGCACCCGCAAGACCTTCCCCAACTACTTCGACG   3598
TaEPSPS-7A1    TGCCAGTCACCATCAGGGACCCTGGATGCACCCGAAAGACCTTCCCCAACTACTTCGACG   3319
TaEPSPS-7D1    TGCCAGTCACCATCAGGGATCCGGGGTGCACCAGAAAGACCTTCCCCAATTACTTCGACG   2691
TaEPSPS-4A1    TGCCAGTCACCATCAGGGACCCCGGGTGCACCCGCAAGACCTTCCCCAACTACTTCGATG   2703
               **** ** ** ******** ** ** ****** * ************** ******** *
                                      <--------------------
                                         R16-D, R16-AB

OsEPSPS        TTCTAAGCACTTTCGTCAGGAACTGAACTGAGCTTTTAAAAGAGTGAGGTCTAGGTTCTG   3658
TaEPSPS-7A1    TGCTAAGCACCTTCGTCAAGAACTAG----------------------------------   3345
TaEPSPS-7D1    TGCTAAGCACCTTCGTCAAGAACTAGATTGGAGAATCTACGGCG----------------   2735
TaEPSPS-4A1    TGCTAAGCACCTTCGTCAAGAACTAG----------------------------------   2729
               * ******** ******* *****
                 <-----------------------
                          R1
```

Additional file 3. Nucleotide sequence alignment and primer positions. ClustalW was used to align the genomic DNA sequences of rice *EPSPS* sequence (*OsEPSPS*) [GenBank:AF413081] with *TaEPSPS-7A1* [KP411547], *TaEPSPS-7D1* [KP411548], and *TaEPSPS-4A1* [KP411549] of *T. aestivum* ‘Louise’. Asterisks indicate fully conserved nucleotides. Plus one (+1) represents the translation start codons of *TaEPSPS-7A1* and *OsEPSPS*. Exon sequences are highlighted in yellow. Labeled arrows indicate the positions of important primers.
