# Supplementary material for: Molecular and phylogenetic characterization of the homoeologous EPSP Synthase genes of allohexaploid wheat, Triticum aestivum (L.)
Source: BMC Genomics. 2015 Oct 23;16:844. doi: 10.1186/s12864-015-2084-1 (PMC4619226; doi:10.1186/s12864-015-2084-1)
Supplement: Additional file 4: — The EPSPS genomic DNA cloning strategy. (PDF 245 kb) [file 12864_2015_2084_MOESM4_ESM.pdf]

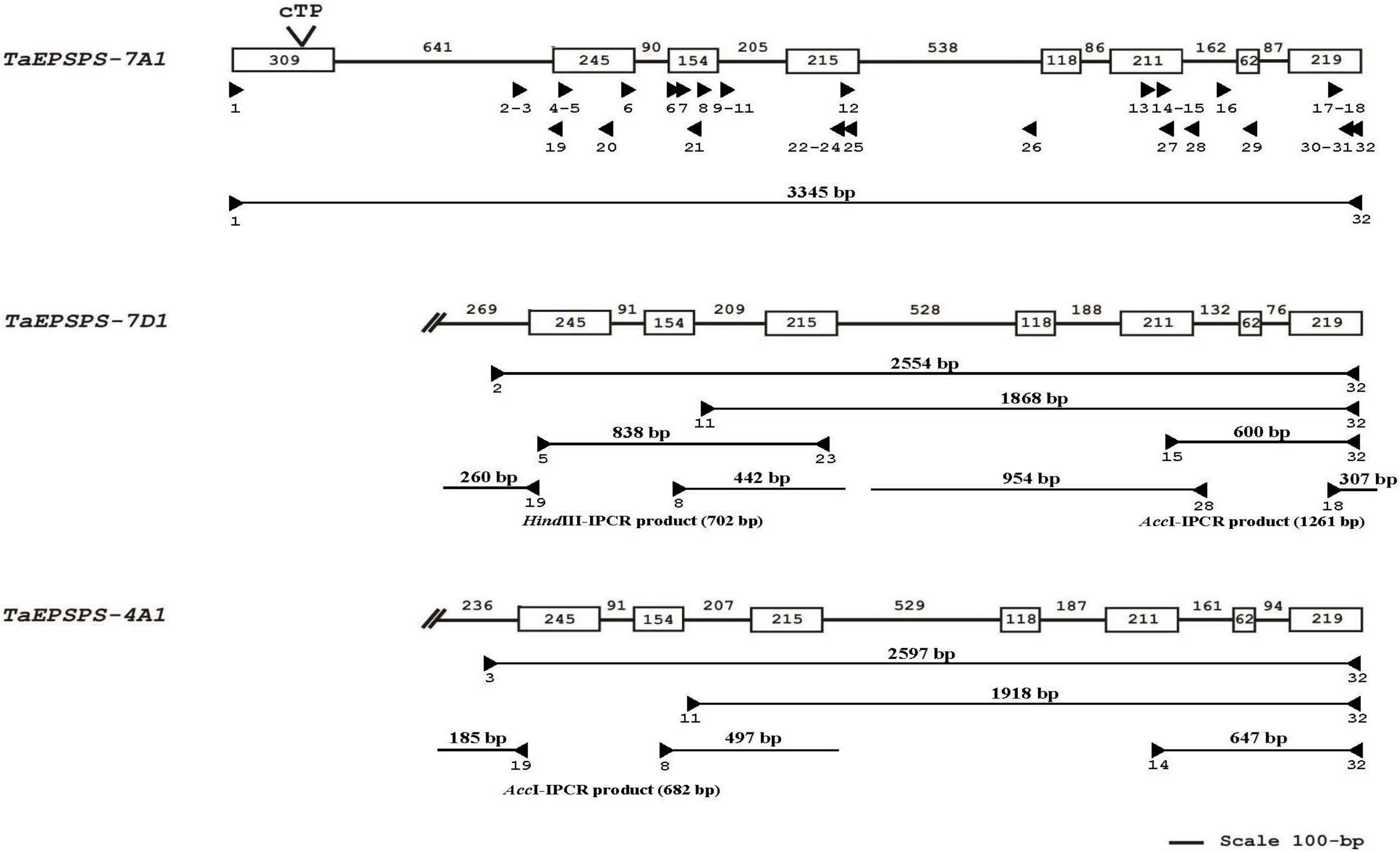

Additional file 4. The *EPSPS* genomic DNA cloning strategy. The genomic sequences of the three wheat *EPSPS* genes were assembled from the sequences of the PCR and IPCR products diagrammed here. Primer positions (triangles) and product sizes (bp) are indicated. The numbers below the triangles indicate primer numbers as shown in Table 1.
