# Supplementary material for: Molecular and phylogenetic characterization of the homoeologous EPSP Synthase genes of allohexaploid wheat, Triticum aestivum (L.)
Source: BMC Genomics. 2015 Oct 23;16:844. doi: 10.1186/s12864-015-2084-1 (PMC4619226; doi:10.1186/s12864-015-2084-1)
Supplement: Additional file 5: — Amino acid sequence alignment of wheat EPSPS. (PDF 47 kb) [file 12864_2015_2084_MOESM5_ESM.pdf]

```
                 +1
OsEPSPS          -MASNAAAAAAVSLDQAVAASAAFSSRKQLRLPAAARGGMRVRVRARGRREAVVVASASS   59
TaEPSPS-7A1      MAMAAAATVAASASSSAVSLDRAAPAHPRRLRMPAARAAHRGAVRLWGPRGAAARATS--   58
TaEPSPS-7D1      ------------------------------------------------------------
TaEPSPS-4A1      ------------------------------------------------------------

                                                              exon 2 start
                                                             ↓
OsEPSPS          SSVAAPAAKAEEIVLQPIREISGAVQLPGSKSLSNRILLLSALSEGTTVVDNLLNSEDVH  119
TaEPSPS-7A1      VAAPAAPAGAEEVVLQPIREISGAVQLPGSKSLSNRILLLSALSEGTTVVDNLLNSEDVH  118
TaEPSPS-7D1      ---------------------------------------------GTTVVDNLLNSEDVH   15
TaEPSPS-4A1      ---------------------------------------------GTTVVDNLLNSEDVH   15
                                                              ***************

OsEPSPS          YMLEALKALGLSVEADKVAKRAVVVGCGGKFPVEKDAKEEVQLFLGNAGTAMRPLTAAVT  179
TaEPSPS-7A1      YMLEALEALGLSVEADKVAKRAVVVGCGGRFPVEKDAKEEVKLFLGNAGTAMRPLTAAVV  178
TaEPSPS-7D1      YMLEALEALGLSVEADKVAKRAVVVGCGGRFPVEKDAKEEVKLFLGNAGTAMRPLTAAVV   75
TaEPSPS-4A1      YMLEALEALGLSVEADKVAKRAVVVGCGGRFPVEKDAKEEVKLFLGNAGTAMRPLTAAVV   75
                 ****** ********************** *********** *****************

OsEPSPS          AAGGNATYVLDGVPRMRERPIGDLVVGLKQLGADVDCFLGTECPPVRVKGIGGLPGGKVK  239
TaEPSPS-7A1      AAGGNATYVLDGVPRMRERPIGDLVVGLQQLGADVDCFLGTNCPPVRINGKGGLPGGKVK  238
TaEPSPS-7D1      AAGGNATYVLDGVPRMRERPIGDLVVGLQQLGADADCFLGTNCPPVRINGKGGLPGGKVK  135
TaEPSPS-4A1      AAGGNATYVLDGVPRMRERPIGDLVVGLQQLGADADCFLGTNCPPVRINGKGGLPGGKVK  135
                 **************************** ***** ****** *****  * *********

OsEPSPS          LSGSISSQYLSALLMAAPLALGDVEIEIIDKLISIPYVEMTLRLMERFGVKAEHSDSWDR  299
TaEPSPS-7A1      LSGSISSQYLSSLLMAAPLALEDVEIEIIDKLISVPYVEMTLKLMEHFGVTAEHSDSWDR  298
TaEPSPS-7D1      LSGSISSQYLSSLLMAAPLALEDVEIEIIDKLISVPYVEMTLKLMERFGVTAEHSDSWDR  195
TaEPSPS-4A1      LSGSISSQYLSSLLMAAPLALEDVEIEIIDKLISVPYVEMTLKLMERFGVTAAHSDSWDR  195
                 *********** ********* ************ ******* *** *** * *******

OsEPSPS          FYIKGGQKYKSPGNAYVEGDASSASYFLAGAAITGGTVTVQGCGTTSLQGDVKFAEVLEM  359
TaEPSPS-7A1      FYIKGGQKYKSPGNAYVEGDASSASYFLAGAAITGGTVTVEGCGTTSLQGDVKFAEVLEM  358
TaEPSPS-7D1      FYIKGGQKYKSPGNAYVEGDASSASYFLAGAAITGGTVTVEGCGTTSLQGDVKFAEVLEM  255
TaEPSPS-4A1      FYIKGGQKYKSPGNAYVEGDASSASYFLAGAAITGGTVTVEGCGTTSLQGDVKFAEVLEM  255
                 **************************************** *******************

OsEPSPS          MGAKVTWTDTSVTVTGPPREPYGKKHLKAVDVNMNKMPDVAMTLAVVALFADGPTAIRDV  419
TaEPSPS-7A1      MGAKVTWTDTSVTVTGPPRQPFGRKHLKAVDVNMNKMPDVAMTLAVVALFADGPTAIRDV  418
TaEPSPS-7D1      MGAKVTWTDTSVTVTGPPRQPFGRKHLKAVDVNMNKMPDVAMTLAVVALFADGPTAIRDV  315
TaEPSPS-4A1      MGAKVTWTDTSVTVTGPPRQPFGRKHLKAVDVNMNKMPDVAMTLAVVALFADGPTAIRDV  315
                 ******************* * * ************************************

OsEPSPS          ASWRVKETERMVAIRTELTKLGASVEEGPDYCIITPPEKLNITAIDTYDDHRMAMAFSLA  479
TaEPSPS-7A1      ASWRVKETERMVAIRTELTKLGATVEEGPDYCIITPPEKLNITAIDTYDDHRMAMAFSLA  478
TaEPSPS-7D1      ASWRVKETERMVAIRTELTKLGATVEEGPDYCIITPPEKLNVTAIDTYDDHRMAMAFSLA  375
TaEPSPS-4A1      ASWRVKETERMVAIRTELTKLGATVEEGPDYCIITPPEKLNVTAIDTYDDHRMAMAFSLA  375
                 *********************** ***************** ******************

OsEPSPS          ACADVPVTIRDPGCTRKTFPNYFDVLSTFVRN  511
TaEPSPS-7A1      ACAEVPVTIRDPGCTRKTFPNYFDVLSTFVKN  510
TaEPSPS-7D1      ACAEVPVTIRDPGCTRKTFPNYFDVLSTFVKN  407
TaEPSPS-4A1      ACAEVPVTIRDPGCTRKTFPNYFDVLSTFVKN  407
                 *** ************************** *
```

Additional file 5. Amino acid sequence alignment of wheat EPSPS. ClustalW was used to align the predicted protein sequences of *TaEPSPS-7A1* [KP411547], *TaEPSPS-7D1* [KP411548], and *TaEPSPS-4A1* [KP411549] from *T. aestivum* with OsEPSPS [AAL06593]. Asterisks indicate fully conserved amino acid residues. Plus one (+1) represents the first amino acid of the TaEPSPS-7A1 protein. The arrow marks the first amino acid of exon 2.
