# Supplementary material for: Molecular and phylogenetic characterization of the homoeologous EPSP Synthase genes of allohexaploid wheat, Triticum aestivum (L.)
Source: BMC Genomics. 2015 Oct 23;16:844. doi: 10.1186/s12864-015-2084-1 (PMC4619226; doi:10.1186/s12864-015-2084-1)
Supplement: Additional file 6: — Conservation of the predicted signal peptide cleavage site. (PDF 69 kb) [file 12864_2015_2084_MOESM6_ESM.pdf]

```
                                   +1
O. sativa           ---------MASNAAAAAAVSLDQAVAASAAFSSRKQLRLPAAARGGMRVRVRARGRREA   51
S. halepense        ------------------------------------------------------------
Z. mays             ------------------------------------------------------------
B. distachyon       ----------MAMAGATTMASKAAAAVSLDRAAGPTASSNLSRRLRMPARSARGLRLRGR   50
TaEPSPS-7A1         ----------MAMAAAATVAASASSSAVSLDRAAPAHPRRLRMPAARAAHRGAVRLWGPR   50
TaEPSPS-7D1         ------------------------------------------------------------
TaEPSPS-4A1         ------------------------------------------------------------

                                   cTP-cleavage site                exon 2 start
                                          ↓                               ↓
O. sativa           VVVASASSSSVAAPAAKAEEIVLQPIREISGAVQLPGSKSLSNRILLLSALSEGTTVVDN  111
S. halepense        ----------------GAEEIVLQPIKEISGTVKLPGSKSLSNRILLLAALSEGTTVVDN   44
Z. mays             ---------------AGAEEIVLQPIKEISGTVKLPGSKSLSNRILLLAALSEGTTVVDN   45
B. distachyon       GGVVLAASVAAPAAPAGAEEVVLQPIREISGAVQLPGSKSLSNRILLLSALSEGTTVVDN  110
TaEPSPS-7A1         GAAARATSVAAPAAPAGAEEVVLQPIREISGAVQLPGSKSLSNRILLLSALSEGTTVVDN  110
TaEPSPS-7D1         ------------------------------------------------------GTTVVDN    7
TaEPSPS-4A1         ------------------------------------------------------GTTVVDN    7

O. sativa           LLNSEDVHYMLEALKALGLSVEADKVAKRAVVVGCGGKFPVEKDAKEEVQLFLGNAGTAM  171
S. halepense        LLNSEDVHYMLGALNTLGLSVEADKVAKRAVAVGCGGKFPVED-AKEEVQLFLGNAGTAM  103
Z. mays             LLNSEDVHYMLGALRTLGLSVEADKAAKRAVVVGCGGKFPVED-AKEEVQLFLGNAGTAM  104
B. distachyon       LLNSEDVHYMLEALKALGLSVEADKVAKRAVVVGCSGKFPVEKDAKEEVQLFLGNAGTAM  170
TaEPSPS-7A1         LLNSEDVHYMLEALEALGLSVEADKVAKRAVVVGCGGRFPVEKDAKEEVKLFLGNAGTAM  170
TaEPSPS-7D1         LLNSEDVHYMLEALEALGLSVEADKVAKRAVVVGCGGRFPVEKDAKEEVKLFLGNAGTAM   67
TaEPSPS-4A1         LLNSEDVHYMLEALEALGLSVEADKVAKRAVVVGCGGRFPVEKDAKEEVKLFLGNAGTAM   67
```

Additional file 6. Conservation of the predicted signal peptide cleavage site. Partial amino acid sequences of the deduced TaEPSPS-7A1, TaEPSPS-7D1, and TaEPSPS-4A1 were aligned with EPSPS of other grasses including *O. sativa* [GenBank:AAL06593], *S. halepense* [AEP26126], *Z. mays* [CAA44974], and *B. distachyon* [XP_003557242]. The locations of the chloroplastic signaling peptide cleavage sites predicted by PredSL are underlined. The exon 2 start site and the predicted cleavage site of chloroplastic signal peptide (cTP) are marked.
